# Comparison and Validation of Some ITS Primer Pairs Useful for Fungal Metabarcoding Studies

**DOI:** 10.1371/journal.pone.0097629

**Published:** 2014-06-16

**Authors:** Michiel Op De Beeck, Bart Lievens, Pieter Busschaert, Stéphan Declerck, Jaco Vangronsveld, Jan V. Colpaert

**Affiliations:** 1 Centre for Environmental Sciences, Hasselt University, Hasselt, Limburg, Belgium; 2 Department of Microbial and Molecular Systems (M2S), Catholic University of Leuven, Antwerp, Belgium; 3 Earth & Life Institute, Catholic University of Louvain, Louvain-la-Neuve, Walloon Brabant, Belgium; University of New South Wales, Australia

## Abstract

Current metabarcoding studies aiming to characterize microbial communities generally rely on the amplification and sequencing of relatively short DNA regions. For fungi, the internal transcribed spacer (ITS) region in the ribosomal RNA (rRNA) operon has been accepted as the formal fungal barcode. Despite an increasing number of fungal metabarcoding studies, the amplification efficiency of primers is generally not tested prior to their application in metabarcoding studies. Some of the challenges that metabarcoding primers should overcome efficiently are the amplification of target DNA strands in samples rich in non-target DNA and environmental pollutants, such as humic acids, that may have been co-extracted with DNA. In the current study, three selected primer pairs were tested for their suitability as fungal metabarcoding primers. The selected primer pairs include two primer pairs that have been frequently used in fungal metabarcoding studies (ITS1F/ITS2 and ITS3/ITS4) and a primer pair (ITS86F/ITS4) that has been shown to efficiently amplify the ITS2 region of a broad range of fungal taxa in environmental soil samples. The selected primer pairs were evaluated in a 454 amplicon pyrosequencing experiment, real-time PCR (qPCR) experiments and *in silico* analyses. Results indicate that experimental evaluation of primers provides valuable information that could aid in the selection of suitable primers for fungal metabarcoding studies. Furthermore, we show that the ITS86F/ITS4 primer pair outperforms other primer pairs tested in terms of *in silico* primer efficiency, PCR efficiency, coverage, number of reads and number of species-level operational taxonomic units (OTUs) obtained. These traits push the ITS86F/ITS4 primer pair forward as highly suitable for studying fungal diversity and community structures using DNA metabarcoding.

## Introduction

Until the late 1980s, microbial ecologists and taxonomists relied on culturing and morphological and physiological characteristics to describe microbial communities and members thereof. In the last two decades, DNA sequencing has revolutionized the way microbial communities are being characterized [1], [2]. In addition, since the introduction of pyrosequencing by Margulies et al. [3], characterization of microbial communities has undergone a second revolution as this technology (used by e.g. Sogin et al. [4] and Buée et al. [5]) enables detailed microbial community characterization at greater sequencing depth than was deemed possible via cloning and Sanger sequencing. A number of next-generation sequencing technologies now enable researchers to identify a large number of organisms from environmental samples using relatively short DNA sequences. This molecular identification method has been termed metabarcoding [6]. Nevertheless, whatever sequencing technology is used, DNA metabarcoding generally depends on the amplification of barcode regions using taxon-specific primers [7]. Such primers need to be universal enough to cover a large group of taxa (e.g. the fungal kingdom), but at the same time have to result in amplicons that are variable enough to efficiently distinguish between closely related species or to identify operational taxonomic units (OTUs) [7], [8]. For fungi and oomycetes, the internal transcribed spacer region (ITS; spanning the ITS1, 5.8S and ITS2 regions) in the ribosomal RNA (rRNA) operon has been recognized as the formal DNA barcoding region [9]–[11].

The full ITS region in fungi has an average length of 500 and 600 base pairs (bp) for ascomycetes and basidiomycetes, respectively, and an average length of 600 bp across all fungal lineages [12]. As current 454 amplicon pyrosequencing (using Roche's Genome Sequencer FLX (GS-FLX) instrument and Titanium chemistry) generates read lengths averaging 450 bp, it is impossible to span the entire ITS region in a single run. Even with recent advances in sequencing technologies that enable sequencing across the entire ITS region, it will probably remain desirable for fungal metabarcoding studies to exclude the 5.8S region of the rRNA operon. The inclusion of conserved regions in DNA sequences is known to increase the risk of chimera formation during PCR [13]. Therefore, generally, either the ITS1 or the ITS2 region is used in ecological studies aiming at the characterization of fungal communities.

Primers that will be used in metabarcoding studies should be able to efficiently amplify their target DNA regions in the presence of high concentrations of non-target DNA and contaminants, such as humic acids, that may have been co-extracted with DNA [14]. Therefore, *in silico* testing of primers is expected to result in an incomplete picture of how primers will behave during amplification of DNA extracted from environmental samples. Comparing the amplification efficiency and robustness of primers used in metabarcoding studies is important because differences in primer efficiency may result in strong biases in favour of more easily amplifiable sequences during PCR reactions, potentially influencing our view on fungal communities [15]–[17]. Moreover, a primer set that covers a large proportion of the species that compose a community of interest and that produces a reliable outcome is desired as ecological metabarcoding studies typically rely on a single primer pair to map microbial diversity.

The most commonly used primers in fungal ecology for sequence-based fungal identification at the species level were published by White et al. [18]: ITS1, ITS2, ITS3 and ITS4, and by Gardes and Bruns [19]: ITS1F and ITS4B. Whereas the primers developed by White et al. [18] had a broad spectrum, ITS1F and ITS4B were developed to be specific for fungi and basidiomycetes respectively [19]. ITS1F is most frequently combined with ITS2 to amplify the ITS1 region of the fungal rRNA operon and ITS3 is usually combined with ITS4 to amplify the ITS2 region. These primer pairs have been used in many branches of mycological research in the past twenty years and are popular tools in recent fungal community studies as well [5], [20]–[24] (also reviewed in Hibbett et al. [25]).

The aim of the current study was to evaluate the amplification efficiency of these established primer pairs and to compare them to a selected primer pair (ITS86F/ITS4) that has been shown to specifically and efficiently amplify ITS sequences from a broad range of fungal taxa in human blood samples as well as in environmental soil samples [26].

## Materials and methods

### Study site and soil sampling

A pioneer pine forest on a stabilised sand dune in the northern part of Limburg, Belgium (Hechtel-Eksel: 51°7′33″N, 5°22′22″E) was selected to obtain samples for this study. The study site is not freely accessible. To gain access to this study site, please contact the responsible authorities (Table S1). The soil in this study site is a dry sandy soil without a litter layer, poor in organic matter and slightly acidic. The average organic carbon content for this site is 0.7% and the average pH is 4.7. The pioneer vegetation at the study site is dominated by young Scots Pine trees (*Pinus sylvestris* L.), mosses and lichens, with only few grasses and heather shrubs (*Calluna vulgaris* (L.) Hull). Tree ages at the time of sampling ranged from one to five years. The region has an average annual rainfall of 800 mm per square meter and the average annual temperature is 10°C (Royal Meteorological Institute, Ukkel, Belgium).

Soil samples for fungal community characterization were collected in November 2009. Samples were collected at a depth of 0 to 20 cm using a soil corer with a diameter of 1 cm. Four replicate soil samples were collected within a distance of ten centimetres from each other for seven sampling locations. Each sampling location was chosen close to a three to five year old pine tree randomly selected in the field. Selected pine trees were at least 20 m apart from each other. The 4 replicate soil samples were pooled for each sampling location, resulting in a total of seven pooled samples. Samples were sealed in plastic bags and tightly closed to prevent desiccation during transportation. Upon arrival in the lab, soil samples were sieved using a 2 mm sieve to homogenize the sample and remove roots, large pieces of organic matter and stones. Samples were subsequently stored at −80°C until DNA was extracted. No protected species were sampled during the study.

### DNA extraction, PCR amplification and pyrosequencing

Approximately 250 mg of soil was used for each DNA extraction. DNA was extracted in quadruplicate from each pooled sample using the UltraClean Soil DNA Isolation Kit according to the manufacturer's protocol (MoBio, Carlsbad, CA, USA). This resulted in four replicates for each of seven pooled soil samples. Subsequently, amplicon libraries were created using barcode-tagged primers for the primer pairs ITS1F/ITS2, ITS3/ITS4 and ITS86F/ITS4 (Table 1). Both forward and reverse primers were synthesized with a tail containing the Roche 454 pyrosequencing adaptors and a sample-specific 10 bp barcode (multiplex identifiers: MIDs) [28] enabling sorting out the obtained sequences after sequencing (Roche Applied Science, Mannheim, Germany). Fusion primers were designed according to the scheme provided in Table S2.

**Table 1:** Primers used in the current study.

Primer name	Primer sequence (5′-3′)	rRNA operon binding site	Reference
ITS1F (F)	CTTGGTCATTTAGAGGAAGTAA	Small subunit	[19]
ITS2 (R)	GCTGCGTTCTTCATCGATGC	5.8S	[18]
ITS3 (F)	GCATCGATGAAGAACGCAGC	5.8S	[18]
ITS4 (R)	TCCTCCGCTTATTGATATGC	Large subunit	[18]
ITS86F (F)	GTGAATCATCGAATCTTTGAA	5.8S	[27]
ITS86R (R)	TTCAAAGATTCGATGATTCAG	5.8S	[26]

Primers are indicated as forward (F) or reverse (R). ITS86R contains a wrong base at the 3′ end. The G should be replaced by a C (see Discussion).

DNA samples were amplified using a Techne TC-5000 thermocycler (Bibby Scientific Limited, Staffordshire, UK) under the following conditions: initial denaturation at 95°C for 2 minutes, followed by 40 cycles of denaturation at 95°C for 30 seconds, annealing at 55°C for 30 seconds and extension at 72°C during 1 minute; a final extension phase was performed at 72°C during 10 minutes. Reactions were carried out in 25 µl reaction volumes using the FastStart High Fidelity PCR System (Roche Applied Science, Mannheim, Germany). Each reaction contained 2.75 µl FastStart 10× reaction buffer, 1.8 mM MgCl, 0.2 mM dNTP mix, 0.4 µM of each primer, 1.25 U FastStart HiFi polymerase and 5 ng template DNA (as measured by a Nanodrop spectrophotometer).

Amplified DNA was cleared from PCR primers and primer dimers using the Agencourt AMpure XP System according to the manufacturer's protocol (Beckman Coulter, Brea, CA, USA). Finally, purified dsDNA was quantified with the Quant-iT PicoGreen dsDNA Assay Kit (Invitrogen, Carlsbad, CA, USA) and a Fluostar Omega plate reader (BMG Labtech, Ortenberg, Germany) and subsequently pooled in equimolar concentrations. The resulting amplicon pool, containing all 84 samples, was sequenced on one fourth of a Pico Titer Plate on a Roche Genome Sequencer FLX System using Titanium chemistry (Roche Applied Science, Mannheim, Germany) according to the manufacturer's instructions.

### Bioinformatics processing

The standard flowgram format (SFF) file that resulted from the interpreted flowgrams was deposited in the NCBI Sequence Read Archive under accession number SRP026207 (SRA, http://www.ncbi.nlm.nih.gov/Traces/sra). From the original SFF file, three separate quality and fasta files were created with a custom Python script according to the three primer pairs used (Table S2). Further analyses were carried out in Mothur 1.31.2 on the individual fastq and fasta files [29]. Quality trimming in Mothur removed reads shorter than 200 bases, reads longer than 600 bases, reads with homopolymers longer than 8 bases and reads containing ambiguous bases. Reads were trimmed when the average Phred quality score dropped below 35 over a window of 50 bases. Next, sequences were compared to each other and duplicate sequences were replaced by a single sequence, while archiving the abundance data of the unique sequences. Subsequently, unique reads were checked for chimeric sequences using the Uchime tool in Mothur followed by their removal from the datasets. Unique reads were aligned with the pairwise alignment tool in Mothur. Finally, species-level OTUs were defined based on a 97% sequence similarity level, which is within the range of intraspecific ITS sequence similarity [30]. In order to further remove potential sequencing errors from the analysis, global singletons (i.e. OTUs represented by only a single sequence over an entire dataset) were removed [24].

Because the primer pairs resulted in different amounts of reads per sample, the number of reads per sample were rarefied to 200 reads per sample. Samples for which less than 200 reads were obtained were removed from the dataset. For ITS1F/ITS2 14 of 28 samples were removed. For ITS3/ITS4 4 samples were removed and for ITS86F/ITS4 no samples were removed. Inter-sample rarefaction curves were constructed based on 10,000 iterations. Subsequently, intra-sample diversity, richness and Good's coverage estimates were calculated in Mothur 1.31.2 based on 10,000 iterations. BLAST searches for a representative sequence of each OTU (as determined by Mothur) were conducted using the PlutoF v2.0 massBLASTer online tool [31]. Reads were blasted against the UNITE [32] and INSD [33] databases. Resulting HTML files were combined with the abundance data obtained in Mothur using a custom Python script. This script also acquired the names of species or genera that resemble Latin binomials with the highest BLAST score, avoiding unidentified OTUs in the databases to be seen as best BLAST hits. Unidentified OTUs were indicated as “not applicable (NA)”.

### Quantitative real-time PCR

To evaluate the performance of the primer pairs amplifying target DNA from a heterogeneous pool of DNA in environmental samples, all primer pairs were tested in a qPCR set-up. A 2-fold dilution series (1∶1 to 1∶64) was made from twelve DNA samples (ranging from 5 ng µl^−1^ to 78 pg µl^−1^, including one no-template control (NTC) for each sample). Amplification was performed in optical 96-well plates using a 7500 Fast Real-Time PCR System (Applied Biosystems, Foster City, CA, USA) and SYBR Green chemistry. PCR conditions were as follows: initial denaturation at 95°C for two minutes, followed by 40 cycles of 95°C (30 s), 55°C (30 s) and 72°C (60 s) and a final extension phase at 72°C for 10 minutes followed by the generation of a dissociation curve to verify amplification specificity. These qPCR conditions were chosen to mimic the PCR conditions used during the PCR step prior to emPCR and amplicon pyrosequencing. Reactions contained 2.5 µL template DNA, 5 µL 2× Fast SYBR Green Master Mix (Applied Biosystems, Foster City, CA, USA), 0.3 µl forward and reverse primers (3.3 µM each) and 1.9 µL nuclease-free H_2_O in a total volume of 10 µL. PCR efficiencies (E) were calculated as E = (10^−1/slope^−1)×100.

To assess a potential PCR-bias at the phylum level, DNA was extracted from 15 pure cultures provided by the Mycothèque de l'Université Catholique de Louvain (BCCM/MUCL) including 5 basidiomycetes (*Lentinula edodes* (MUCL 44827), *Agrocybe praecox* (MUCL 46727), *Coniophora marmorata* (MUCL 39471), *Suillus luteus* (UH-Slu-LM8-n1) and *Antrodia vaillantii* (MUCL 54533)), 5 ascomycetes (*Cladosporium cladosporioides* (MUCL 53652), *Cryptosporiopsis radicicola* (MUCL 53485), *Monilinia laxa* (MUCL 30841), *Arthroderma otae* (MUCL 39756) and *Galactomyces geotrichum* (MUCL 52377)), 2 glomeromycetes (*Rhizophagus clareus* (MUCL 46238) and *Rhizophagus* sp. (MUCL 41833)) and 3 zygomycetes (*Mortierella verticillata* (MUCL 9658), *Absidia corymbifera* (MUCL 38907) and *Mucor hiemalis* (MUCL 15439), also see Table S4). DNA was extracted from cultures using the DNeasy Plant Mini Kit according to the manufacturer's instructions (Qiagen, Venlo, Netherlands). DNA concentrations extracted from pure cultures used for qPCR ranged from 5 ng µl^−1^ to 20 ng µl^−1^. PCR bias at the phylum level was tested according to the qPCR protocol described above.

### *In silico* evaluation of primer pairs

To evaluate the primer-to-target mismatches *in silico*, primers were tested with PrimerProspector 1.0.1 [34] against sequences downloaded from NCBI. Three sets of sequences were downloaded from NCBI containing only full-length fungal 5.8S, 18S and 28S sequences. Duplicate sequences were removed using Mothur 1.31.2. ITS1F was tested against 3,748 18S rDNA sequences. ITS2, ITS3 and ITS86F were tested against 4,421 5.8S rDNA sequences. ITS4 was tested against 4,270 28S rDNA sequences. For comparison, also all primers investigated by Ihrmark et al. [35] and Toju et al. [36] were tested [18]–[19], [27], [35]–[38]. All tests were performed as described by Walters et al. [34] using standard settings. Primer scores were calculated based on the following formula: weighted score = non-3′ mismatches ×0.40+3′ mismatches ×1.00+non-3′ gaps ×1.00+3′ gaps ×3.00. An additional penalty score of 3.00 was assigned if the final 3′ base of a primer had a mismatch with its target sequence [34].

### Statistical analysis

Statistical analyses were conducted in R 2.13.0 (The R Foundation for Statistical Computing, Vienna, Austria). Normal distributions of the residuals of models were checked with the Shapiro-Wilk test, while homoscedasticity of variances was analysed using either Bartlett's or the Fligner-Killeen test. Depending on the distribution of the estimated parameters, either ANOVA or the Kruskal-Wallis Rank Sum Test was used to check for significant differences in variances of parameters. Two-by-two comparisons were conducted using either Tukey's Honest Significant Differences tests or Pairwise Wilcoxon Rank Sum Tests. Poisson corrections were implemented for abundance data. Distributions of ratios were compared with Pearson's Chi-squared tests. Non-metric multi-dimensional scaling (NMDS) was performed using the Vegan 2.0 - 8 package in R.

## Results

### Parametrical analysis of 454 amplicon pyrosequencing data

For the three tested primer pairs, GS-FLX sequencing of the amplicon libraries generated a total of 151,650 reads. For a read to be successfully assigned to a sample, we required that both the forward and the reverse MIDs and primers were identified in a read with no more than one erroneous base in the MIDs and no more than two erroneous bases in the primer sequences. Based on the primer and MID sequences, 65,133 reads were assigned to their respective sample and 86,517 reads remained unassigned. The average length of reads assigned to either ITS1F/ITS2, ITS3/ITS4 or ITS86F/ITS4 prior to quality checking and trimming was 314, 331 and 369 bp respectively (excluding primers). The average read length of the unassigned reads was 116 bp (including primers, data not shown).

**Figure 1:**
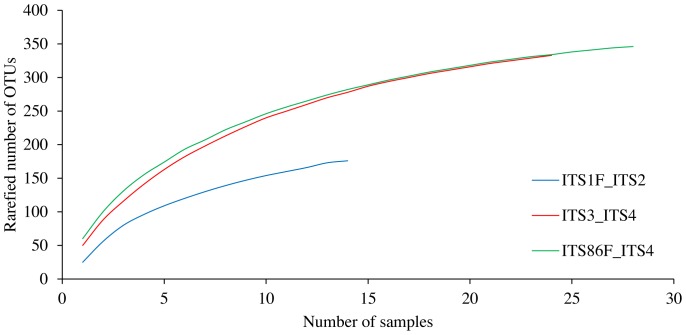
Rarefaction curves for each of the three primer pairs used in this study: ITS1F/ITS2, ITS3/ITS4 and ITS86F/ITS4. In these graphs, the number of samples is plotted against the rarefied number of operational taxonomic units (OTUs) that were created based on a 97% sequence similarity cut-off value.

Rarefaction curves were constructed showing the rarefied number of OTUs defined at a 97% sequence similarity threshold relative to the number of samples (Fig. 1). These results indicate that, on average, a higher OTU richness and a better coverage of the fungal community can be expected for the ITS86F/ITS4 and ITS3/ITS4 primer pairs. The lowest OTU richness and coverage was predicted for the ITS1F/ITS2 primer pair. As most rarefaction curves tended towards saturation, the sequencing depth was assumed to be sufficient to retrieve the most abundant fungal OTUs in analysed soil samples that are detectable by the respective primers and 454 amplicon pyrosequencing.

**Figure 2:**
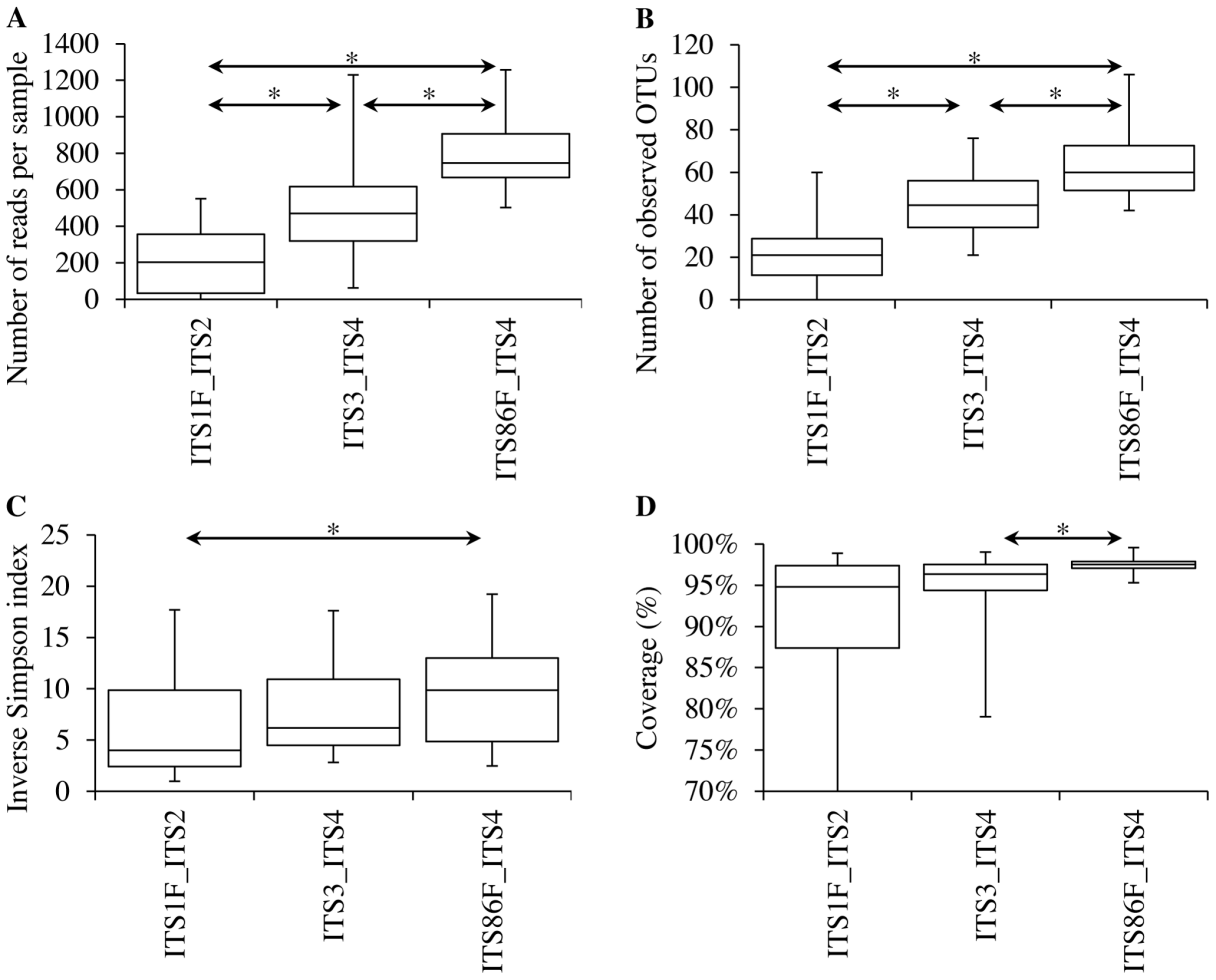
Parametrical comparison between the three primer pairs used in this study (ITS1F/ITS2, ITS3/ITS4 and ITS86F/ITS4). A. Average number of sequences obtained after quality trimming. B. Average number of operational taxonomic units (OTUs), based on a 97% sequence similarity cut-off value. C. Average inverse Simpson index. D. Average Good's coverage. Averages were calculated across replicates (four) and samples (seven) for each primer pair. Differences at the 95% significance level are indicated with an asterisk “*”.

To compare primer pair performance in the 454 amplicon pyrosequencing experiment, averages of the number of reads were calculated across replicates (four replicates per sample) and samples (seven samples) for each primer pair. The average number of reads per sample obtained by ITS1F/ITS2, ITS3/ITS4 and ITS86F/ITS4 after quality trimming differed significantly (p<0.01) and primer pairs yielded on average (± standard error) 356 (±26), 523 (±43) and 797 (±34) high quality reads per sample, respectively (Fig. 2A). The average number of OTUs found for each primer pair at a 97% sequence similarity threshold (observed OTU richness) also differed significantly (p<0.01). The highest OTU richness was observed for ITS86F/ITS4 with an average of 62 OTUs per sample (min = 42; max = 106). ITS1F/ITS2 yielded on average 32 OTUs per sample (min = 15; max = 60), whereas ITS3/ITS4 resulted in an average of 50 OTUs per sample (min = 27; max = 76) (Fig. 2B). Diversity was estimated with the inverse Simpson index. The inverse Simpson index differed significantly between ITS86F/ITS4 and ITS1F/ITS2, whereas with ITS1F/ITS2 a lower diversity was found than with ITS86F/ITS4 (p = 0.04). However, no significant differences were found between ITS3/ITS4 and ITS1F/ITS2 or between ITS3/ITS4 and ITS86F/ITS4 (p = 0.31 and p = 0.53, respectively) (Fig. 2C). The average Good's coverage per sample obtained for ITS1F/ITS2 was 96.8% (min = 93.8%, max = 98.9%), whereas the average Good's coverage obtained for ITS3/ITS4 and ITS86F/ITS4 was 96.5% (min = 93.2%, max = 99.0%) and 97.5% (min = 95.3%, max = 99.6%) respectively (Fig. 2D). Significant differences in Good's coverage were found between ITS3/ITS4 and ITS86F/ITS4 (p<0.01). However, no significant differences were found between ITS1F/ITS2 and ITS3/ITS4 (p = 0.81) or between ITS1F/ITS2 and ITS86F/ITS4 (p = 0.31).

### Community similarity compared between primer pairs

**Figure 3:**
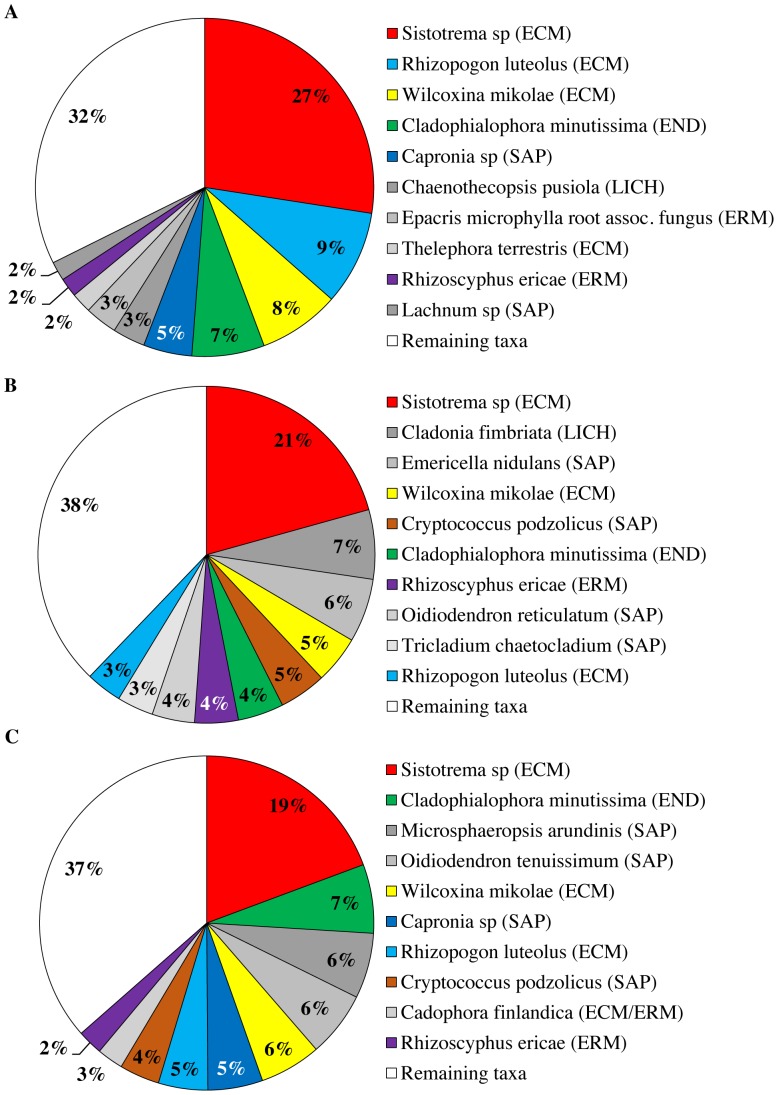
Relative abundance for the top ten most abundant species-level operational taxonomic units (OTUs), based on a 97% sequence similarity cut-off value, obtained for each of the three primer pairs studied (ITS1F/ITS2, ITS3/ITS4 and ITS86F/ITS4). Reads that did not result in a BLAST hit against the UNITE or INSD databases were indicated as “not applicable (NA)”. Ecological functions of OTUs are indicated between brackets behind the OTU identities (ECM: ectomycorrhizal, ERM: ericoid mycorrhizal, SAP: saprotrophic, LICH: lichenized, END: endophytic). OTUs not belonging to the top ten most abundant OTUs were pooled in the category “Remaining taxa”. OTUs that appear exclusively in a single chart are indicated in grayscale. OTUs that can be found in multiple pie charts are indicated in colour. OTU abundance scores were averaged across replicates (four) and samples (seven). A. ITS1F/ITS2. B. ITS3/ITS4. C. ITS86F/ITS4.

**Figure 4:**
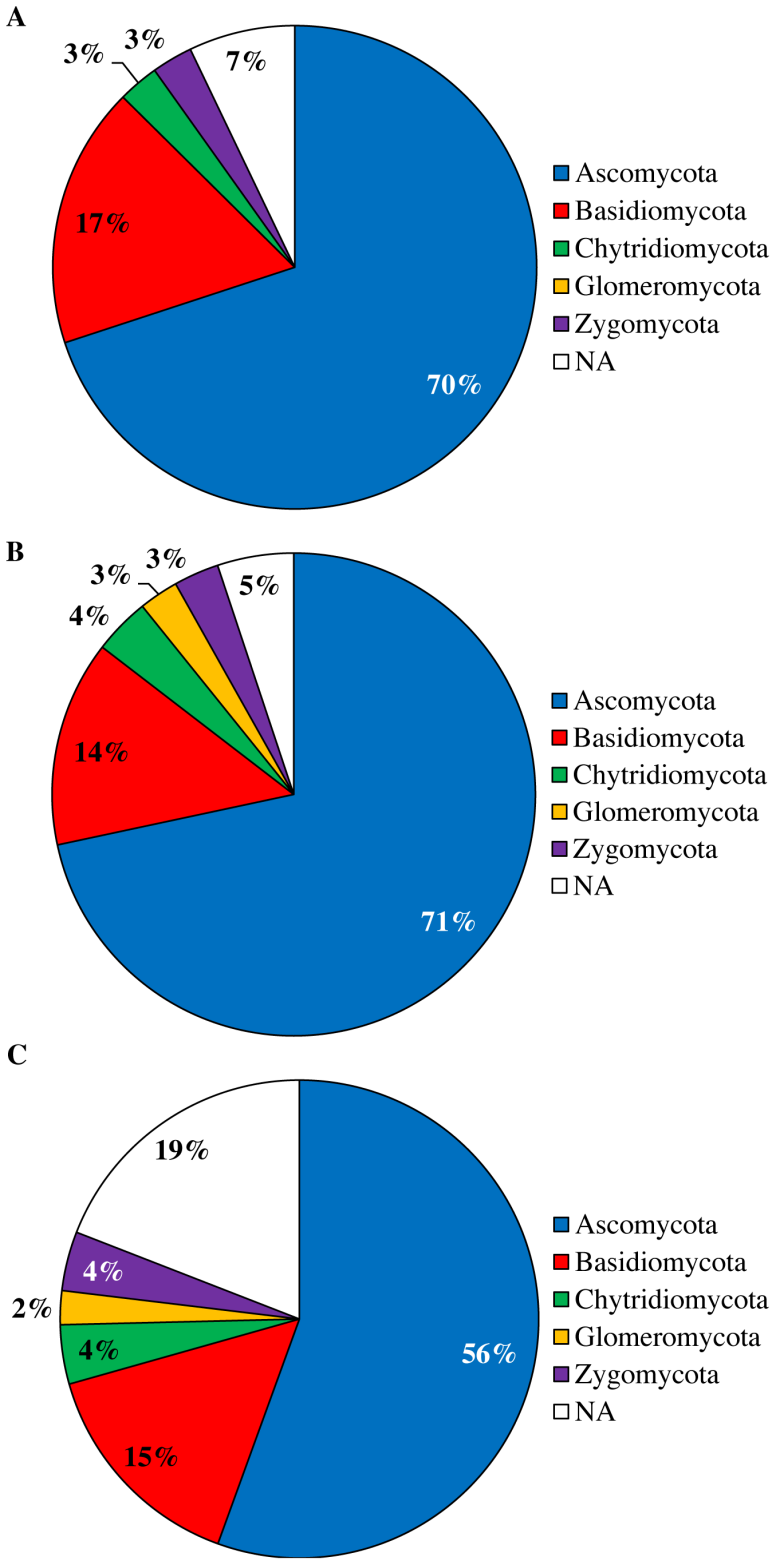
Relative number of OTUs belonging to different fungal phyla. OTUs that could not be assigned to a phylum were grouped together under “not applicable (NA)”. Averages were calculated across replicates (four) and samples (seven). A. ITS1F/ITS2. B. ITS3/ITS4. C. ITS86F/ITS4.

To compare the fungal community characterized with ITS1F/ITS2, ITS3/ITS4 and ITS86F/ITS4 at the species and phylum level, a representative sequence of each OTU (as selected by Mothur) was blasted against the UNITE and INSD databases using the massBLASTer tool in PlutoF v2.0 [31]. Relative frequency distributions of the obtained species-level OTUs and phyla were analysed with chi-squared tests for the different primer pairs, based on the average abundances across replicates (four) and samples (seven). Representative reads of OTUs that could not be coupled to an accession of either the UNITE or INSD databases were considered as unidentified OTUs (indicated as not applicable “NA” in Table S3). A total of 51 unidentified OTUs were found of which 50 were found with ITS86F/ITS4 and 1 with ITS3/ITS4. BLAST scores and corresponding E-values for all OTUs can be found in Table S3. At the species level, differences were observed between the fungal communities identified by the three primer pairs studied (p<0.01). To give an idea of the fungal communities identified by each primer pair, pie charts displaying the top ten most abundant OTUs were constructed covering 68%, 62% and 64% of all sequences obtained with ITS1F/ITS2, ITS3/ITS4 and ITS86F/ITS4, respectively (Fig. 3). Using the ITS1F/ITS2 primer pair (targeting the ITS1 region) a total of 183 OTUs across all samples were observed, with the most abundant OTUs corresponding to *Sistotrema* sp. Fr. (27%), *Rhizopogon luteolus* Fr. (9%), *Wilcoxina mikolae* (Chin S. Yang & H.E. Wilcox) Chin S. Yang & Korf (8%), *Cladophialophora minutissima* M.L. Davey & Currah (7%), and *Capronia* sp. Sacc. (5%) (Fig. 3A). The primer pairs ITS3/ITS4 and ITS86F/ITS4 (targeting the ITS2 region) identified 333 and 346 OTUs across all samples, respectively. In line with ITS1F/ITS2, the fungal communities identified with ITS3/ITS4 and ITS86F/ITS4 were also dominated by *Sistotrema* sp. (21-19%), but the subdominant OTUs were not exactly the same (Fig. 3B,C). Interesting to note is that the soil samples were dominated by ectomycorrhizal and ericoid mycorrhizal fungi and mycobionts from lichens. Based on field observations, we assumed that the fungal community in the pioneer forest that was sampled in this study would be relatively species poor compared to old forest soils [5] and that biotrophic fungi would dominate over saprotrophic ones. These assumptions were confirmed by all three primer pairs (Fig. 3). At the phylum level, differences in community composition were found between all primer pairs tested (p<0.01 for all comparisons) (Fig. 4). Nevertheless, the majority of OTUs identified by all tested primer pairs belonged to the phyla Ascomycota (56% to 71%), followed by Basidiomycota (14% to 17%). A minority of OTUs identified, belonged to the Zygomycota (3% to 4%), Chytridiomycota (3% to 4%) and Glomeromycota (0% to 3%) (Fig. 4).

### Repeatability of metabarcoding results

The repeatability of the molecular identification of fungal OTUs from environmental samples was compared between the three tested primer pairs to assess their experimental robustness. Replicates of samples were compared for each primer pair using NMDS with Bray-Curtis dissimilarities. In this analysis, samples with a similar OTU-composition will have smaller Bray-Curtis distances than samples with more dissimilar OTU compositions. In general, for all three primer pairs, replicates from the same sample grouped closely together (especially for ITS3/ITS4) (Fig. S1). Hence, the results of molecular identification of fungal OTUs are fairly consistent between replicated samples using the current experimental set-up. In order to test the possibility that some OTUs are missed in metabarcoding analyses based on the amplification and sequencing of target DNA from a single DNA extraction, results from the four replicated DNA extractions of the same sample were compared (Fig. S2). This assessment was performed for the four most abundant OTUs, representing *Sistotrema* sp., *Rhizopogon luteolus*, *Cladophialophora minutissima* and *Wilcoxina mikolae*. From Fig. S2, it is clear that in some replicated extractions of the same sample abundant OTUs can be missed. These results indicate that PCR amplification and sequencing can best be performed on multiple DNA extractions from the same environmental sample that are pooled prior to PCR in order to obtain an accurate picture of a fungal community.

### Efficiency of primer pairs studied

To test the amplification efficiency of the three primer pairs in a heterogeneous pool of DNA (environmental sample) a qPCR experiment was conducted. More specifically, a 2-fold dilution series, ranging from 1∶1 to 1∶64 dilutions of twelve randomly selected DNA samples were amplified with randomly selected ITS1F/ITS2, ITS3/ITS4 and ITS86F/ITS4 primers with MIDs and 454 adaptors attached. For ITS1F/ITS2, exponential amplification was obtained between 24 and 32 PCR cycles for ten out of twelve samples (data not shown). For two samples no exponential amplification phase was obtained within 40 cycles with this primer pair. ITS3/ITS4 showed exponential amplification after 22 to 36 cycles for all twelve samples, whereas ITS86F/ITS4 already showed an exponential amplification phase after 20 to 31 cycles for all samples (data not shown). Average PCR efficiencies (± standard error) were calculated to be 76% (±4%) for ITS3/ITS4, 82% (±5%) for ITS1F/ITS2 and 97% (±6%) for ITS86F/ITS4 (Table 2).

**Table 2:** Average PCR amplification efficiencies obtained for twelve environmental DNA samples using quantitative real-time PCR.

Primer pair	ITS1F_ITS2	ITS3_ITS4	ITS86F_ITS4
Average (%)	82	76	97
Standard error (%)	4	5	6
Minimum (%)	64	67	78
Maximum (%)	97	103	120

### Phylum-level PCR bias

**Figure 5:**
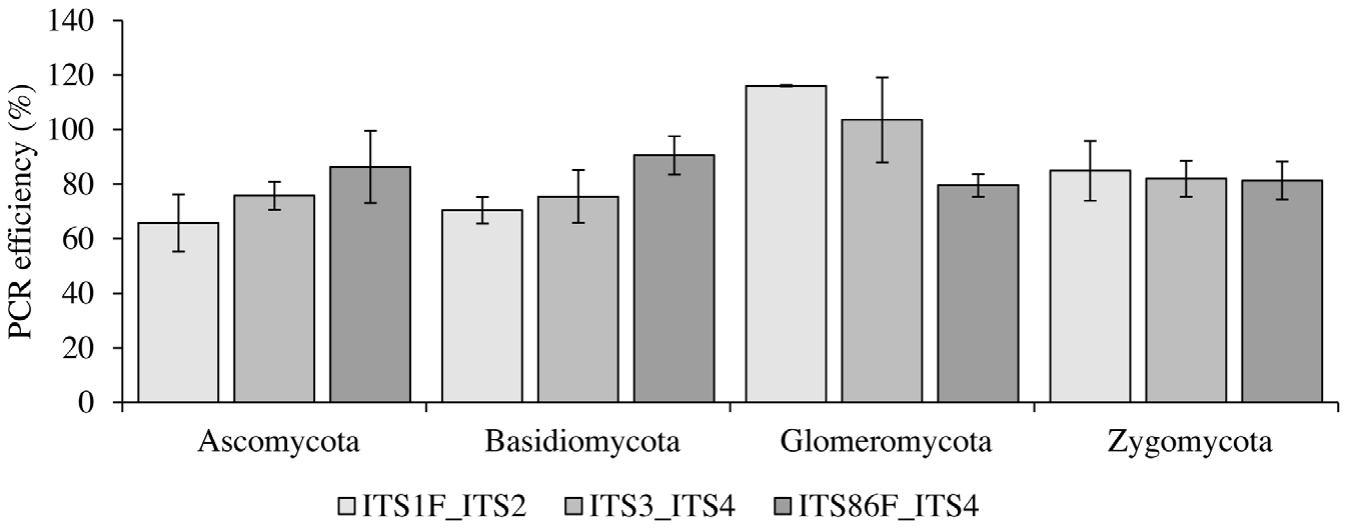
Phylum-level PCR bias assessed using qPCR. Average PCR efficiencies were calculated for each phylum using 5 basidiomycetes, 5 ascomycetes, 2 glomeromycetes and 3 zygomycetes. Error bars represent standard errors. No significant differences between primer pairs and phyla were found at the 95% significance level.

qPCR amplification efficiency did not significantly differ between primer pairs tested (ITS1F/ITS2, ITS3/ITS4 and ITS86F/ITS4), nor between phyla (Ascomycota, Basidiomycota, Glomeromycota and Zygomycota) (Fig. 5). Two-way ANOVA resulted in p = 0.14 for phylum and p = 0.59 for primer pair. Primer pair - rDNA target combinations with poor PrimerProspector scores tended to have slightly lower PCR efficiencies, but these differences were not significant. Strains of species used for this experiment and PCR efficiencies can be found in Table S4 and Table S5, respectively.

### *In silico* evaluation of primers

In a final analysis, the primer-to-target mismatches of the three primer pairs used in this study were evaluated with PrimerProspector [34]. PrimerProspector was used to calculate a score for each primer based on mismatches between primers and target DNA sequences. The closer the score of a primer is to 0, the fewer mismatches were detected between primers and target sequences. The average scores (± standard error) for primers used in our study were: ITS1F = 4.55 (±0.05), ITS2 = 0.70 (±0.03), ITS3 = 0.58 (±0.03), ITS4 = 3.96 (±0.04) and ITS86F = 0.52 (±0.02) (Table 3). Moreover, it was found that 44% of the tested sequences had a mismatch with the last base at the 3′ end of primer ITS1F. This particular mismatch between the last base at the 3′ end of a primer sequence and a target sequence occurred with only 9%, 4%, 16% and 3% of the tested sequences for ITS2, ITS3, ITS4 and ITS86F respectively (Table 3). For comparison, also the primers suggested by Ihrmark et al. [35] and Toju et al. [36] were tested with PrimerProspector. Also in this analysis, ITS86F was found to have the best primer score of all tested primers (Table S6).

**Table 3:** Results of *in silico* testing of primers using PrimerProspector 1.0.1 [34].

Primer	Number of sequences tested	3′ end base mismatch (%)	Average score ± SE
ITS1F (F)	3748	44%	4.6±0.05
ITS2 (R)	4421	9%	0.7±0.03
ITS3 (F)	4421	4%	0.6±0.03
ITS4 (R)	4270	16%	4.0±0.04
ITS86F (F)	4421	3%	0.0±0.00

Primers are indicated as forward (F) or reverse (R). Average PrimerProspector scores are shown ± standard errors (SE).

## Discussion

Amplification and sequencing of short, standard DNA regions (metabarcoding) is becoming an increasingly popular tool for the characterization of fungal communities. Nevertheless, in most fungal metabarcoding studies, primers are generally used without being tested for their efficiency to amplify heterogeneous DNA pools, which may affect our view on studied fungal communities. Whereas the most commonly used primers in fungal metabarcoding studies were designed in the 90 s for species identification of a limited number of focal species, environmental metabarcoding studies generally aim to characterize diverse communities in environmental samples. Hence, primers used for fungal metabarcoding should be able to amplify a broad range of target DNA sequences in a sample that is also rich in non-target DNA and that may contain environmental contaminants [39]. Even though recent efforts have resulted in new primers that could amplify a large proportion of target fungal DNA sequences [35], [36], an experimental evaluation of PCR efficiency and primer performance should be performed on real environmental samples.

Initially, also ITS1F/ITS86R was included in our study design, but this primer pair was discarded from the study as no amplification was obtained in exploratory PCR and gel-electrophoresis tests. A plausible explanation for this failure can be found in the fact that the reverse primer (ITS86R) used and reported by Turenne et al. [27] and Vancov and Keen [26] contains an incorrect base at the 3′ end of the primer sequence. In order to be the perfect reverse complement of ITS86F, the sequence of ITS86R should be 5′-TTCAAAGATTCGATGATTCAC-3′, and not 5′-TTCAAAGATTCGATGATTCAG-3′ as reported. GS-FLX sequencing of the amplicon pool resulted in 151,650 raw reads prior to quality trimming. Of these reads, 65,133 were assigned to their respective sample and 86,517 reads remained unassigned. The unassigned reads were investigated manually revealing that the majority were primer sequences probably resulting from primer dimers in our sequenced amplicon pool. Most likely, these primer dimers were not sufficiently removed during post-PCR clean-up steps.

Rarefaction curves were constructed for each primer pair (Fig. 1). These rarefaction curves indicate that the highest rarefied OTU richness and best coverage of the fungal community can be expected for the ITS86F/ITS4 and ITS3/ITS4 primer pairs. The average observed number of reads and the average observed number of OTUs (derived from these reads at a 97% sequence identity cut-off) indeed were highest for the ITS86F/ITS4 primer pair (797 reads and 62 OTUs on average per sample) and the ITS3/ITS4 primer pair (523 reads, 50 OTUs) and were much lower for the ITS1F/ITS2 primer pair (356 reads and 32 OTUs) (Fig. 2). The average observed diversity per sample, estimated by the inverse Simpson index, did not differ between ITS3/ITS4 and ITS86F/ITS4, but was significantly lower for ITS1F/ITS2 (Fig. 2). Overall, the low number of OTUs per sample found in the current study, are in sharp contrast with the more than 1000 OTUs per gram of forest soil found by Buée et al. [5] based on amplification with the ITS1F/ITS2 primer pair. This difference in richness may be explained by the fact that pioneer forests probably contain relatively fewer fungal species compared to old forest soils [5]. Additionally, overestimation or underestimation of species richness can also originate from data handling and analysis [40]. Based on the *in silico* performance and high Good's coverage calculated for ITS86F, it can be expected that the 62 OTUs found on average per sample by the ITS86F/ITS4 primer pair is close to the real species richness for the pioneer ecosystem growing on stabilised sand dunes which was studied here. The 50 OTUs per sample found by ITS3/ITS4 and the 32 OTUs found by ITS1F/ITS2, are probably underestimations due to a more narrow primer spectrum and/or lower PCR efficiencies. The fact that a high Good's coverage was found for the ITS1F/ITS2 primer pair despite a low observed OTU richness indicates that this primer pair is unable to multiply the ITS1 region of a large number of fungi. This is also supported by the *in silico* analysis. In this analysis, ITS1F was shown to have the poorest primer score of 4.6 and its sequence was shown to have a mismatch at the final base at the 3′ end of the primer (having a detrimental effect on amplification efficiency [41]) with no less than 44% of the tested fungal sequences (Table 3). The large number of mismatches between the ITS1F primer and its target sequences was previously also addressed by Bellemain et al. [42] and Ihrmark et al. [35]. In comparison, the ITS4 primer was given a score of 4.0 and was found to have a primer-to-target mismatch at the 3′ end of the primer with only 16% of the tested sequences. For the ITS2, ITS3 and ITS86F primers a score of 0.7, 0.6 and 0.0 was obtained respectively (Table 3). These primers were shown to have a mismatch at the 3′ end of the primer with only 9%, 4% and 4% of the tested sequences, respectively (Table 3), illustrating their broad amplification potential. Furthermore, our *in silico* analyses indicated that the primers suggested by Ihrmark et al. [35] and Toju et al. [36] had more mismatches to their respective target sequences than ITS86F.

To test how these parametrical differences would translate to amplification efficiency during PCR amplification preceding emulsion PCR (emPCR) and pyrosequencing, a first qPCR experiment was conducted. To this end, DNA was extracted from 12 soil samples and amplified with the same primer pairs used in the pyrosequencing experiment. The calculated PCR efficiencies were 82% for ITS1F/ITS2, 76% for ITS3/ITS4 and 97% for ITS86F/ITS4 (Table 2). From these PCR efficiencies, it is clear that ITS86F/ITS4 amplified its target ITS regions with greater efficiency than the other two primer pairs. Contrary to our expectations from the *in silico* analysis, ITS3/ITS4 obtained a lower efficiency than the ITS1F/ITS2 primer pair. This could be explained by the fact that also other factors determine the amplification efficiency of PCR reactions beside binding and dissociation of primers to their target DNA sequences. Such factors include the temperature-dependent properties of target DNA sequences and primer sequences in the PCR mixture, the temperature-dependent behaviour of the used polymerase enzyme mixtures, the use of ROX as an endogenous reference dye, etc. [39]. Alternatively, the range of target sequences that ITS1F and ITS2 may bind to during PCR amplification is smaller, but the sequences that do get bound by these primers are amplified efficiently.

To see whether differences in amplification efficiency between primer pairs would also be reflected in the identities of the OTUs identified in the 454 amplicon pyrosequencing experiment, a representative read for each OTU was blasted against the UNITE and INSD databases and the BLAST hits with the highest score and a species or genus name were used to reconstruct the fungal community for each primer pair (Fig. 3). According to all three primer pairs, the soil fungal community was dominated by an OTU corresponding to *Sistotrema* sp. Additionally, all primer sets produced a number of OTUs that were commonly identified by all primer pairs (Fig. 3). The community identified by the three tested primer pairs still differed significantly, however. These differences confirm the finding that targeting either the ITS1 or the ITS2 region may result in different pictures of the fungal communities at the OTU level, as was previously assessed by both *in silico* [43] and sequencing studies [40], [44]. In addition, it was found that primers targeting the same ITS region do not necessarily result in the same OTU composition (Fig. 3), highlighting the importance of primer choice in a given study. However, it needs to be noted that in comparative studies, it has been shown that an ecological signal can be much stronger than the differences in community composition originating from primer choice [44].

At the phylum level, significant differences between ITS1F/ITS2, ITS3/ITS4 and ITS86F/ITS4 were found as well (Fig. 4). Although in varying proportions, all three primer pairs identified more OTUs belonging to ascomycetes (70%, 71% and 56% respectively) than basidiomycetes (17%, 14% and 15%), but also Chytridiomycota (3%, 4%, 4%), Glomeromycota (0%, 3% and 2%) and Zygomycota (3%, 3% and 4%) were detected (Fig. 4). This might suggest that more ascomycetes were present in the soil at the time of investigation. However, amplification of DNA from ascomycetes may be favoured relative to amplification of DNA from basidiomycetes as the ITS sequences for ascomycetes are generally shorter than basidiomycete ITS sequences (this is especially true for the ITS2 region [12]) and amplification of shorter DNA fragments is favoured during PCR. Whereas in previous *in silico* analyses indeed a phylum-level bias was expected for some of the primers used [42], no such bias was found in the current study based on experimental data derived from qPCR of DNA extracted from 15 fungal species belonging to the major fungal phyla (Fig. 5).

Whatever the aim of a metabarcoding study, results obtained from metabarcoding need to be reliable. To assess the repeatability of the fungal metabarcoding experiment, we analysed four replicate DNA extractions of seven soil samples separately. The analysis of all replicates of samples revealed that replicated analysis of the same sample with a specific primer pair generally results in similar fungal community compositions (Fig. S1). This is especially true for the ITS3/ITS4 and ITS86F/ITS4 primer pairs as their replicated samples clustered nicely together. However, this is less true for the ITS1F/ITS2 primer pair, where replicates of samples tend to have greater projected Bray-Curtis distances (Fig. S1). Moreover, we have shown that it is possible to miss certain OTUs, even abundant ones, when one sequences amplicon pools that are constructed from a single DNA extraction (Fig. S2). It is therefore advisable to extract DNA from environmental samples in multiple replicates, pool the eluates and perform PCR and sequencing on the DNA from the mixed eluate. This observation is in line with other studies performed previously, demonstrating that at least three replicated extractions are required to obtain a DNA pool that is representative for the microbial community present in a given soil sample [45], [46].

Apart from the technical issues that were addressed in this study, our data also provided a glimpse at the fungal community present in the studied site. Based on field observations of above-ground basidiocarps, we assumed that pioneer pine forests in the Campine region in Belgium are dominated by biotrophic species (mostly lichens, ectomycorrhizal and ericoid mycorrhizal fungi) over saprotrophic species. All three primer pairs confirmed this assumption, but they found different fungal OTUs to be dominant. According to the results obtained with ITS1F/ITS2, the fungal community in the studied site was dominated by OTUs corresponding to *Sistotrema* sp. (27%), followed by *Rhizopogon luteolus* (9%), *Wilcoxina mikolae* (8%) and *Cladophialophora minutissima* (7%) (Fig. 3) [47]. These OTUs were also found to be very important members of the studied community according to ITS3/ITS4 and ITS86F/ITS4 as they appeared in the top ten of the most abundant OTUs found by both primer pairs, although in varying proportions (Fig. 3). *Sistotrema* sp., likely an important member of our studied ecosystem, was recently shown to be polyphyletic, containing both ectomycorrhizal and saprotrophic taxa [48]. The reads that were found in the current study correspond to *Sistotrema* strains that were sampled from ectomycorrhizal root tips of *Pinus contorta* Dougl. growing on coastal sand dunes [49]. This genus provides a fine example of the power of molecular tools, such as DNA metabarcoding, to draw attention to ecologically important, cryptic fungal species. Based on field observations alone (basidiocarps observations and root tip morphotying), we never expected this genus to be so abundant in this pioneer ecosystem.

### Concluding remarks

In many fungal metabarcoding studies universal primers from previous phylogenetic or ecological studies are used without first performing an evaluation of their spectrum and performance for high-throughput sequencing, potentially resulting in a biased description of fungal communities. Whereas *in silico* PCR analyses on sequences retrieved from sequence databases may suggest promising primers [35], [36], we showed that an experimental set-up to evaluate their usefulness in practice provides complementary information on the actual performance of the primers for high-throughput sequencing of environmental samples. Indeed, here we demonstrated that the choice of primers has a significant impact on how fungal communities are translated into OTU communities and subsequent data analysis. As such, before setting up large-scale sequencing experiments, we recommend to first test a number of promising primer pairs, e.g. selected with *in silico* analyses, under real PCR conditions for a subset of the samples under investigation. In case an in-depth characterization of a fungal community is desired, the use of more than one primer pair is advisable. We also showed that quantitative real-time PCR, evaluating the efficiency of selected primer pairs, may help in selecting the most efficient primer pairs. After all, using primer pairs that are not very efficient in amplifying DNA from an environmental sample will undoubtedly result in a low number of reads, and consequently in biased community descriptions.

In this study, the primer pair ITS86F/ITS4, which amplifies the ITS2 region of the fungal rRNA operon, was shown to be the most suitable primer pair for the characterization of fungal communities with metabarcoding. This primer pair not only resulted in superior amplification efficiency leading to a significantly higher number of reads, but also yielded a high number of OTUs belonging to different phyla. In addition, this primer pair resulted in a robust amplification reaction for the broadest range of samples and across replicated extractions.

## Supporting Information

Figure S1 **NMDS comparing community dissimilarities (based on Bray-Curtis distances) between replicates of samples.**
(PDF)

Figure S2 **Bar charts displaying the relative number of reads identified by the three primer pairs studied (ITS1F/ITS2, ITS3/ITS4 and ITS86F/ITS4) assigned to one of the four most abundant OTUs.**
(PDF)

Table S1 **Contact addresses for access to the study site.**
(PDF)

Table S2 **Primer design used in the current 454 amplicon pyrosequencing experiment.**
(PDF)

Table S3 **Complete list of OTUs identified in the 454 amplicon pyrosequencing experiment and corresponding BLAST scores and E-values.**
(PDF)

Table S4 **Species used to assess PCR bias of the studied primer pairs (ITS1F/ITS2, ITS3/ITS4 and ITS86F/ITS4).**
(PDF)

Table S5 **PCR efficiencies for the amplification of the ITS region of the fungal rRNA operon with the studied primer pairs (ITS1F/ITS2, ITS3/ITS4 and ITS86F/ITS4).**
(PDF)

Table S6 **PrimerProspector scores of primers used in the current study and investigated by Ihrmark et al.** [35] **and Toju et al.** [36] **.**
(PDF)

## References

[1] Stahl DA, Lane DJ, Olsen GJ, Pace NR (1984) Analysis of hydrothermal vent-associated symbionts by ribosomal-RNA sequences. Science 224: 409–411. 17741220 10.1126/science.224.4647.409

[2] Hugenholtz P, Pace NR (1996) Identifying microbial diversity in the natural environment: a molecular phylogenetic approach. Trends Biotechnol 14: 190–197. 8663938 10.1016/0167-7799(96)10025-1

[3] Margulies M, Egholm M, Altman WE, Attyia S, Bader JS, et al (2005) Genome sequencing in microfabricated high-density picolitre reactors. Nature 437: 376–380. 16056220 10.1038/nature03959 PMC1464427

[4] Sogin ML, Morrison HG, Huber JA, Welch DW, Huse SM, et al (2006) Microbial diversity in the deep sea and the underexplored “rare biosphere”. Proc Natl Acad Sci USA 103: 12115–12120. 16880384 10.1073/pnas.0605127103 PMC1524930

[5] Buée M, Reich M, Murat C, Morin E, Nilsson RH, et al (2009) 454 Pyrosequencing analyses of forest soils reveal an unexpectedly high fungal diversity. New Phytol 184: 449–456. 19703112 10.1111/j.1469-8137.2009.03003.x

[6] Taberlet P, Coissac E, Pompanon F, Brochmann C, Willerslev E (2012) Towards next-generation biodiversity assessment using DNA metabarcoding. Mol Ecol 8: 2045–2050. 10.1111/j.1365-294X.2012.05470.x 22486824

[7] Hebert PDN, Cywinska A, Ball SL, deWaard JR (2003) Biological identifications through DNA barcodes. Proc R Soc B - Biol Sci 270: 313–321. 10.1098/rspb.2002.2218 PMC1691236 12614582

[8] Justé A, Thomma BPHJ, Lievens B (2008) Recent advances in molecular techniques to study microbial communities in food-associated matrices and processes. Food Microbiol 25: 745–761. 18620966 10.1016/j.fm.2008.04.009

[9] Seifert KA (2009) Progress towards DNA barcoding of fungi. Mol Ecol Resour 9: 83–89. 10.1111/j.1755-0998.2009.02635.x 21564968

[10] Begerow D, Nilsson H, Unterseher M, Maier W (2010) Current state and perspectives of fungal DNA barcoding and rapid identification procedures. Appl Microbiol Biotechnol 87: 99–108. 20405123 10.1007/s00253-010-2585-4

[11] Schoch CL, Seifert KA, Huhndorf S, Robert V, Spouge JL, et al (2012) Nuclear ribosomal internal transcribed spacer (ITS) region as a universal DNA barcode marker for Fungi. Proc Natl Acad Sci USA 109: 6241–6246. 22454494 10.1073/pnas.1117018109 PMC3341068

[12] Porter TM, Golding GB (2011) Are similarity- or phylogeny-based methods more appropriate for classifying internal transcribed spacer (ITS) metagenomic amplicons? New Phytol 192: 775–782. 21806618 10.1111/j.1469-8137.2011.03838.x

[13] Haas BJ, Gevers D, Earl AM, Feldgarden M, Ward DV, et al (2011) Chimeric 16S rRNA sequence formation and detection in Sanger and 454-pyrosequenced PCR amplicons. Genome Res 21: 494–504. 21212162 10.1101/gr.112730.110 PMC3044863

[14] Kosch TA, Summers K (2013) Techniques for minimizing the effects of PCR inhibitors in the chytridiomycosis assay. Mol Ecol Resour 13: 230–236. 23241137 10.1111/1755-0998.12041

[15] Polz MF, Cavanaugh CM (1998) Bias in template-to-product ratios in multitemplate PCR. Appl Environ Microbiol 64: 3724–3730. 9758791 10.1128/aem.64.10.3724-3730.1998 PMC106531

[16] Jumpponen A (2007) Soil fungal communities underneath willow canopies on a primary successional glacier forefront: rDNA sequence results can be affected by primer selection and chimeric data. Microb Ecol 53: 233–246. 17106807 10.1007/s00248-004-0006-x

[17] Engelbrektson A, Kunin V, Wrighton KC, Zvenigorodsky N, Chen F, et al (2010) Experimental factors affecting PCR-based estimates of microbial species richness and evenness. ISME J 4: 642–647. 20090784 10.1038/ismej.2009.153

[18] White TJ, Bruns TD, Lee SB, Taylor JW (1990) Amplification and direct sequencing of fungal ribosomal RNA genes for phylogenetics. In: Innis MA, Gelfand DH, Sninsky JJ, White TJ, editors. PCR protocols: a guide to methods and applications. United States: Academic Press. pp. 315–322.

[19] Gardes M, Bruns TD (1993) ITS primers with enhanced specificity for basidiomycetes – application to the identification of mycorrhizae and rusts. Mol Ecol 2: 113–118. 8180733 10.1111/j.1365-294x.1993.tb00005.x

[20] Jumpponen A, Jones KL (2009) Massively parallel 454 sequencing indicates hyperdiverse fungal communities in temperate *Quercus macrocarpa* phyllosphere. New Phytol 184: 438–448. 19674337 10.1111/j.1469-8137.2009.02990.x

[21] Amend AS, Seifert KA, Bruns TD (2010) Quantifying microbial communities with 454 pyrosequencing: does read abundance count? Mol Ecol 19: 5555–5565. 21050295 10.1111/j.1365-294X.2010.04898.x

[22] Ghannoum MA, Jurevic RJ, Mukherjee PK, Cui F, Sikaroodi M, et al (2010) Characterization of the oral fungal microbiome (mycobiome) in healthy individuals. PLoS Pathog 6: e1000713. 20072605 10.1371/journal.ppat.1000713 PMC2795202

[23] Jumpponen A, Jones KL, Mattox D, Yaege C (2010) Massively parallel 454-sequencing of fungal communities in *Quercus* spp. ectomycorrhizas indicates seasonal dynamics in urban and rural sites. Mol Ecol 19: 41–53. 20331769 10.1111/j.1365-294X.2009.04483.x

[24] Tedersoo L, Nilsson RH, Abarenkov K, Jairus T, Sadam A, et al (2010) 454 Pyrosequencing and Sanger sequencing of tropical mycorrhizal fungi provide similar results but reveal substantial methodological biases. New Phytol 188: 291–301. 20636324 10.1111/j.1469-8137.2010.03373.x

[25] Hibbett DS, Ohman A, Glotzer D, Nuhn M, Kirk P, et al (2011) Progress in molecular and morphological taxon discovery in Fungi and options for formal classification of environmental sequences. Fungal Biol Rev 25: 38–47.

[26] Vancov T, Keen B (2009) Amplification of soil fungal community DNA using the ITS86F and ITS4 primers. FEMS Microbiol Lett 296: 91–96. 19459948 10.1111/j.1574-6968.2009.01621.x

[27] Turenne CY, Sanche SE, Hoban DJ, Karlowsky JA, Kabani AM (1999) Rapid identification of fungi by using the ITS2 genetic region and an automated fluorescent capillary electrophoresis system. J Clin Microbiol 37: 1846–1851. 10325335 10.1128/jcm.37.6.1846-1851.1999 PMC84966

[28] Carlsen T, Aas AB, Lindner D, Vrålstad T, Schumacher T, et al (2012) Don't make a mista(g)ke: is tag switching an overlooked source of error in amplicon pyrosequencing studies? Fungal Ecol 5: 747–749.

[29] Schloss PD, Westcott SL, Ryabin T, Hall JR, Hartmann M, et al (2009) Introducing mothur: open-source, platform-independent, community-supported software for describing and comparing microbial communities. Appl Environ Microbiol 75: 7537–7541. 19801464 10.1128/AEM.01541-09 PMC2786419

[30] Blaalid R, Kumar S, Nilsson RH, Abarenkov K, Kirk PM, et al (2013) ITS1 versus ITS2 as DNA metabarcodes for fungi. Mol Ecol Resour 13: 218–224. 23350562 10.1111/1755-0998.12065

[31] Abarenkov K, Tedersoo L, Nilsson RH, Vellak K, Saar I, et al (2010) PlutoF - a web based workbench for ecological and taxonomic research, with an online implementation for fungal ITS sequences. Evol Bioinform 6: 189–196.

[32] Kõljalg U, Larsson KH, Abarenkov K, Nilsson RH, Alexander IJ, et al (2005) UNITE: a database providing web-based methods for the molecular identification of ectomycorrhizal fungi. New Phytol 166: 1063–1068. 15869663 10.1111/j.1469-8137.2005.01376.x

[33] Nakamura Y, Cochrane G, Karsch-Mizrachi I (2013) The international nucleotide sequence database collaboration. Nucl Acids Res 41: D21–D24. 23180798 10.1093/nar/gks1084 PMC3531182

[34] Walters WA, Caporaso JG, Lauber CL, Berg-Lyons D, Fierer N, et al (2011) PrimerProspector: de novo design and taxonomic analysis of PCR primers. Bioinformatics 27: 1159–1161. 21349862 10.1093/bioinformatics/btr087 PMC3072552

[35] Ihrmark K, Bödeker ITM, Cruz-Martinez K, Friberg H, Kubartova A, et al (2012) New primers to amplify the fungal ITS2 region - evaluation by 454-sequencing of artificial and natural communities. FEMS Microbiol Ecol 82: 666–677. 22738186 10.1111/j.1574-6941.2012.01437.x

[36] Toju H, Tanabe AS, Yamamoto S, Sato H (2012) High-coverage ITS primers for the DNA-based identification of ascomycetes and basidiomycetes in environmental samples. PLoS One 7: e40863. 22808280 10.1371/journal.pone.0040863 PMC3395698

[37] Martin KJ, Rygiewicz PT (2005) Fungal-specific PCR primers developed for analysis of the ITS region of environmental DNA extracts. BMC Microbiol 5: 28. 15904497 10.1186/1471-2180-5-28 PMC1156903

[38] Egger KN (1995) Molecular analysis of ectomycorrhizal fungal communities. Canadian J Bot 73: 1415–1415.

[39] Kennedy S, Oswald N (2011) PCR troubleshooting and optimization: the essential guide. United Kingdom: Caister Academic Press. 235 p.

[40] Bazzicalupo AL, Bálint M, Schmitt I (2013) Comparison of ITS1 and ITS2 rDNA in 454 sequencing of hyperdiverse fungal communities. Fungal Ecol 6: 102–109.

[41] Lefever S, Pattyn F, Hellemans J, Vandesompele J (2013) Single-nucleotide polymorphisms and other mismatches reduce performance of quantitative PCR assays. Clin Chem 59: 1470–1480. 24014836 10.1373/clinchem.2013.203653

[42] Bellemain E, Carlsen T, Brochmann C, Coissac E, Taberlet P, et al (2010) ITS as an environmental DNA barcode for fungi: an *in silico* approach reveals potential PCR biases. BMC Microbiol 10: 1–9. 20618939 10.1186/1471-2180-10-189 PMC2909996

[43] Nilsson RH, Ryberg M, Abarenkov K, Sjökvist E, Kristiansson E (2009) The ITS region as a target for characterization of fungal communities using emerging sequencing technologies. FEMS Microbiol Lett 296: 97–101. 19459974 10.1111/j.1574-6968.2009.01618.x

[44] Monard C, Gantner S, Stenlid J (2013) Utilizing ITS1 and ITS2 to study environmental fungal diversity using pyrosequencing. FEMS Microbiol Ecol 84: 165–175. 23176677 10.1111/1574-6941.12046

[45] Feinstein LM, Sul WJ, Blackwood CB (2009) Assessment of bias associated with incomplete extraction of microbial DNA from soil. Appl Environ Microbiol 75: 5428–5433. 19561189 10.1128/AEM.00120-09 PMC2725469

[46] Lindahl BD, Nilsson RH, Tedersoo L, Abarenkov K, Carlsen T, et al (2013) Fungal community analysis by high-throughput sequencing of amplified markers - a user's guide. New Phytol 199: 288–299. 23534863 10.1111/nph.12243 PMC3712477

[47] Davey ML, Currah RS (2007) A new species of *Cladophialophora* (hyphomycetes) from boreal and montane bryophytes. Mycol Res 111: 106–116. 17169546 10.1016/j.mycres.2006.10.004

[48] Münzenberger B, Schneider B, Nilsson RH, Bubner B, Larsson KH, et al (2012) Morphology, anatomy, and molecular studies of the ectomycorrhiza formed axenically by the fungus *Sistotrema* sp. (Basidiomycota). Mycol Prog 11: 817–826.

[49] Ashkannejhad S, Horton TR (2006) Ectomycorrhizal ecology under primary succession on coastal sand dunes: interactions involving *Pinus contorta*, suilloid fungi and deer. New Phytol 169: 345–354. 16411937 10.1111/j.1469-8137.2005.01593.x

