# Supplementary material for: Comparison and Validation of Some ITS Primer Pairs Useful for Fungal Metabarcoding Studies
Source: PLoS One. 2014 Jun 16;9(6):e97629. doi: 10.1371/journal.pone.0097629 (PMC4059633; doi:10.1371/journal.pone.0097629)
Supplement: Figure S1 — NMDS comparing community dissimilarities (based on Bray-Curtis distances) between replicates of samples. (PDF) [file pone.0097629.s001.pdf]

Supporting Information Figure S1

Results of non-metric multi-dimensional scaling (NMDS) comparing community dissimilarities (based on Bray-Curtis distances) between each replicate (n = 4) of a sample (n = 7). Replicates are indicated by the same icon colour within a graph. Replicates with more similar communities are plotted more closely together than more dissimilar replicates. A. ITS1F/ITS2. B. ITS3/ITS4. C. ITS86F/ITS4

A

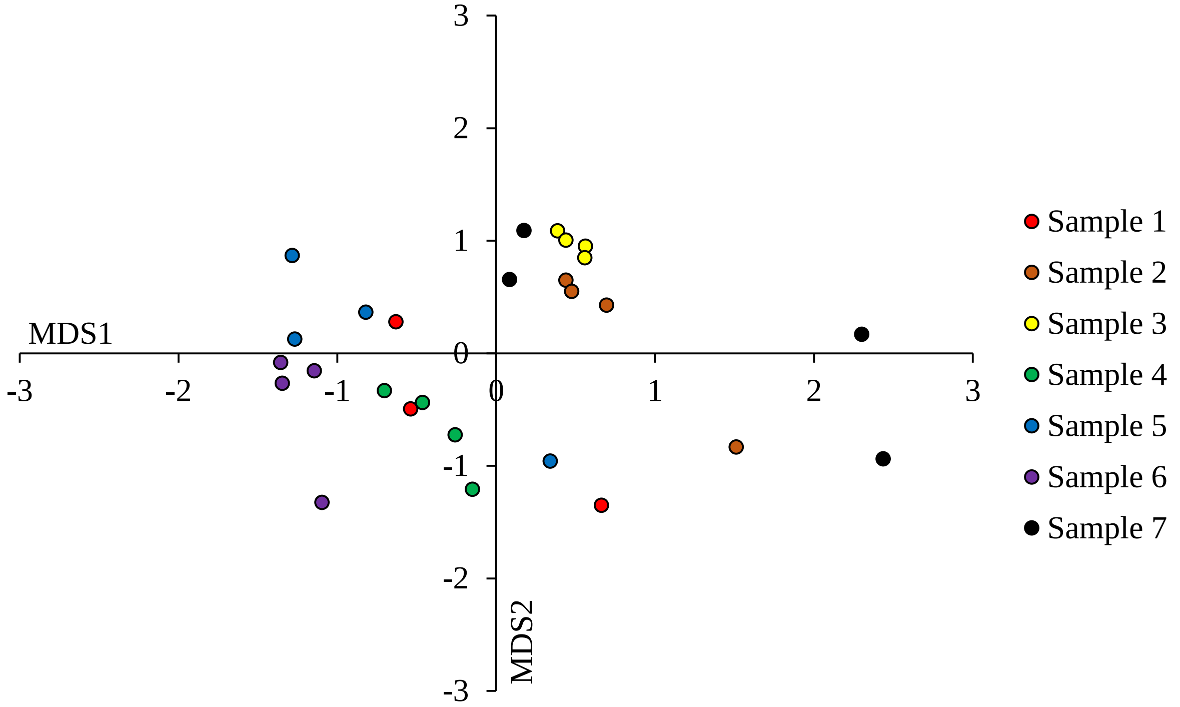

B

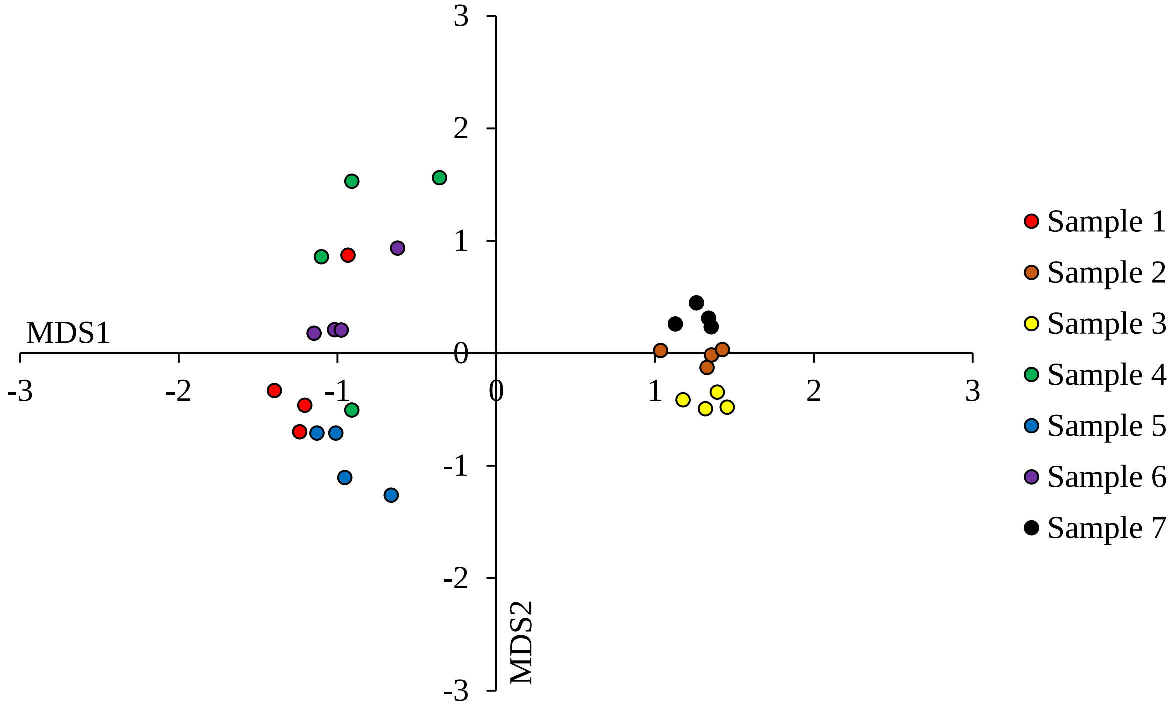
3
2
1
0
-1
-2
-3
MDS1
-3
-2
-1
0
1
2
3
MDS2
Sample 1
Sample 2
Sample 3
Sample 4
Sample 5
Sample 6
Sample 7

C

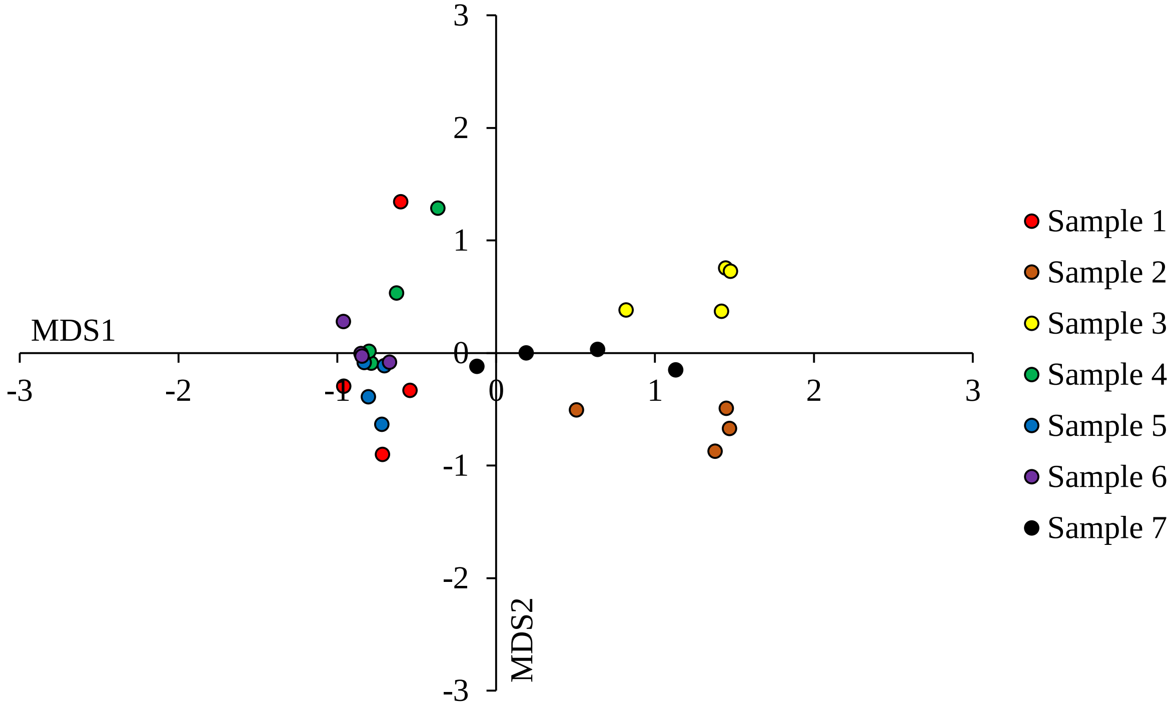
3
2
1
0
-1
-2
-3
MDS1
-3
-2
-1
0
1
2
3
MDS2
Sample 1
Sample 2
Sample 3
Sample 4
Sample 5
Sample 6
Sample 7
