# Supplementary material for: Comparison and Validation of Some ITS Primer Pairs Useful for Fungal Metabarcoding Studies
Source: PLoS One. 2014 Jun 16;9(6):e97629. doi: 10.1371/journal.pone.0097629 (PMC4059633; doi:10.1371/journal.pone.0097629)
Supplement: Figure S2 — Bar charts displaying the relative number of reads identified by the three primer pairs studied (ITS1F/ITS2, ITS3/ITS4 and ITS86F/ITS4) assigned to one of the four most abundant OTUs. (PDF) [file pone.0097629.s002.pdf]

Supporting Information Figure S2

Bar charts displaying the relative amount of reads that were assigned to a specific species-level operational taxonomic unit (OTU) by each primer pair used in this study (ITS1F/ITS2, ITS3/ITS4 and ITS86F/ITS4). Only the four most abundant species-level OTUs were displayed. The x-axes display all replicates and samples separately, where replicates appear as bars in the same colour and samples as different colours. The y-axes show the amount of reads found by a specific primer pair for one replicate of a sample relative (%) to the total amount of reads found for that OTU across all primer pairs, replicates and samples. A. *Sistotrema* sp. B. *Cladophialophora minutissima* C. *Wilcoxina mikolae* and D. *Rhizopogon luteolus*

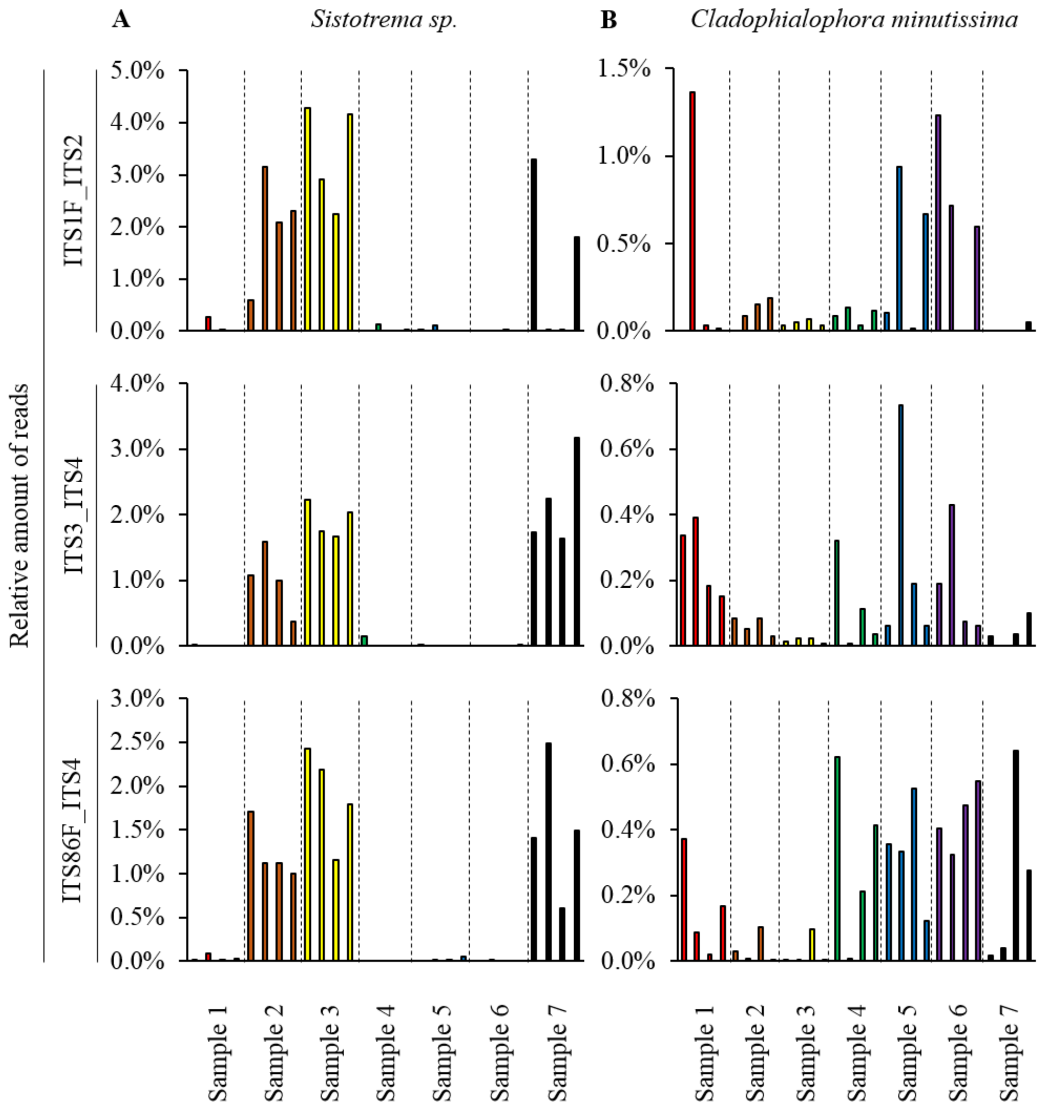

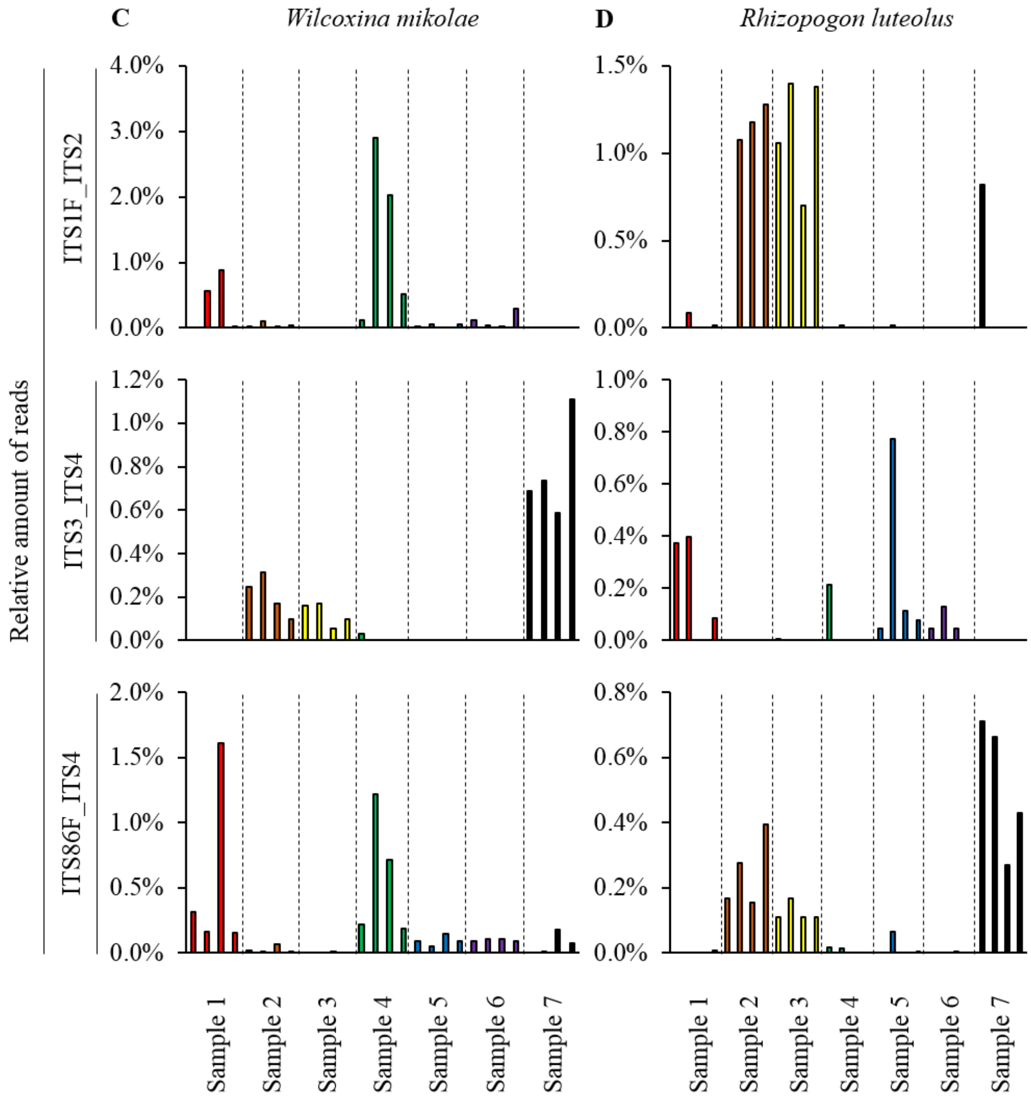

C
Wilcoxina mikolae
D
Rhizopogon luteolus
Relative amount of reads
ITS1F_ITS2
ITS3_ITS4
ITS86F_ITS4
4.0%
3.0%
2.0%
1.0%
0.0%
1.2%
1.0%
0.8%
0.6%
0.4%
0.2%
0.0%
2.0%
1.5%
1.0%
0.5%
0.0%
1.5%
1.0%
0.5%
0.0%
1.0%
0.8%
0.6%
0.4%
0.2%
0.0%
0.8%
0.6%
0.4%
0.2%
0.0%
Sample 1
Sample 2
Sample 3
Sample 4
Sample 5
Sample 6
Sample 7
