# Supplementary material for: Comparison and Validation of Some ITS Primer Pairs Useful for Fungal Metabarcoding Studies
Source: PLoS One. 2014 Jun 16;9(6):e97629. doi: 10.1371/journal.pone.0097629 (PMC4059633; doi:10.1371/journal.pone.0097629)
Supplement: Table S1 — Contact addresses for access to the study site. (PDF) [file pone.0097629.s003.pdf]

**Supporting Information Table S1**

To gain access to the study site, please contact:

**Lt. Col. Van Reckem**
Military Domain Camp Beverloo
Kwartier 4 RCI
Koningin Louisa-Marialaan 3
BE - 3970 Leopoldsburg

**Lt. Col. Fuchs**
**Comm. E. Gorrens**
Military Domain Camp Beverloo
Bureel Planning
Koninklijk Park
BE - 3970 Leopoldsburg
Phone: +32 (0) 11 39 53 35
Phone: +32 (0) 11 39 53 36
E-mail: eddy.gorrens@mil.be
