# Supplementary material for: Comparison and Validation of Some ITS Primer Pairs Useful for Fungal Metabarcoding Studies
Source: PLoS One. 2014 Jun 16;9(6):e97629. doi: 10.1371/journal.pone.0097629 (PMC4059633; doi:10.1371/journal.pone.0097629)
Supplement: Table S2 — Primer design used in the current 454 amplicon pyrosequencing experiment. (PDF) [file pone.0097629.s004.pdf]

Supporting Information Table S2

The following table displays the study design used in the 454 amplicon pyrosequencing experiment. Samples (n = 7), replicates of samples (n = 4) and used primers are indicated. Primers used for pyrosequencing are displayed as their respective components (454 adapter, multiplex identifier (MID) and fungal ITS primers).

Sample	Replicate	Amplicon	Forward primer*			Reverse primer*		
			454 adapter	MID	Fungal ITS primer	454 adapter	MID	Fungal ITS primer
Sample 1	Replicate 1	Amplicon 1	A	MID12	ITS1F	B	MID8	ITS2
		Amplicon 2	A	MID12	ITS3	B	MID8	ITS4
		Amplicon 3	A	MID12	ITS86F	B	MID8	ITS4
	Replicate 2	Amplicon 4	A	MID9	ITS1F	B	MID12	ITS2
		Amplicon 5	A	MID9	ITS3	B	MID12	ITS4
		Amplicon 6	A	MID9	ITS86F	B	MID12	ITS4
	Replicate 3	Amplicon 7	A	MID8	ITS1F	B	MID7	ITS2
		Amplicon 8	A	MID8	ITS3	B	MID7	ITS4
		Amplicon 9	A	MID8	ITS86F	B	MID7	ITS4
	Replicate 4	Amplicon 10	A	MID8	ITS1F	B	MID8	ITS2
		Amplicon 11	A	MID8	ITS3	B	MID8	ITS4
		Amplicon 12	A	MID8	ITS86F	B	MID8	ITS4
Sample 2	Replicate 1	Amplicon 13	A	MID12	ITS1F	B	MID9	ITS2
		Amplicon 14	A	MID12	ITS3	B	MID9	ITS4
		Amplicon 15	A	MID12	ITS86F	B	MID9	ITS4
	Replicate 2	Amplicon 16	A	MID9	ITS1F	B	MID7	ITS2
		Amplicon 17	A	MID9	ITS3	B	MID7	ITS4
		Amplicon 18	A	MID9	ITS86F	B	MID7	ITS4
	Replicate 3	Amplicon 19	A	MID8	ITS1F	B	MID9	ITS2
		Amplicon 20	A	MID8	ITS3	B	MID9	ITS4
		Amplicon 21	A	MID8	ITS86F	B	MID9	ITS4
	Replicate 4	Amplicon 22	A	MID9	ITS1F	B	MID8	ITS2
		Amplicon 23	A	MID9	ITS3	B	MID8	ITS4
		Amplicon 24	A	MID9	ITS86F	B	MID8	ITS4

Sample 3	Replicate 1	Amplicon 25	A	MID9	ITS1F	B	MID9	ITS2
		Amplicon 26	A	MID9	ITS3	B	MID9	ITS4
		Amplicon 27	A	MID9	ITS86F	B	MID9	ITS4
	Replicate 2	Amplicon 28	A	MID10	ITS1F	B	MID12	ITS2
		Amplicon 29	A	MID10	ITS3	B	MID12	ITS4
		Amplicon 30	A	MID10	ITS86F	B	MID12	ITS4
	Replicate 3	Amplicon 31	A	MID10	ITS1F	B	MID7	ITS2
		Amplicon 32	A	MID10	ITS3	B	MID7	ITS4
		Amplicon 33	A	MID10	ITS86F	B	MID7	ITS4
	Replicate 4	Amplicon 34	A	MID8	ITS1F	B	MID10	ITS2
		Amplicon 35	A	MID8	ITS3	B	MID10	ITS4
		Amplicon 36	A	MID8	ITS86F	B	MID10	ITS4
Sample 4	Replicate 1	Amplicon 37	A	MID10	ITS1F	B	MID8	ITS2
		Amplicon 38	A	MID10	ITS3	B	MID8	ITS4
		Amplicon 39	A	MID10	ITS86F	B	MID8	ITS4
	Replicate 2	Amplicon 40	A	MID9	ITS1F	B	MID10	ITS2
		Amplicon 41	A	MID9	ITS3	B	MID10	ITS4
		Amplicon 42	A	MID9	ITS86F	B	MID10	ITS4
	Replicate 3	Amplicon 43	A	MID10	ITS1F	B	MID9	ITS2
		Amplicon 44	A	MID10	ITS3	B	MID9	ITS4
		Amplicon 45	A	MID10	ITS86F	B	MID9	ITS4
	Replicate 4	Amplicon 46	A	MID10	ITS1F	B	MID10	ITS2
		Amplicon 47	A	MID10	ITS3	B	MID10	ITS4
		Amplicon 48	A	MID10	ITS86F	B	MID10	ITS4
Sample 5	Replicate 1	Amplicon 49	A	MID12	ITS1F	B	MID10	ITS2
		Amplicon 50	A	MID12	ITS3	B	MID10	ITS4
		Amplicon 51	A	MID12	ITS86F	B	MID10	ITS4
	Replicate 2	Amplicon 52	A	MID11	ITS1F	B	MID7	ITS2
		Amplicon 53	A	MID11	ITS3	B	MID7	ITS4
		Amplicon 54	A	MID11	ITS86F	B	MID7	ITS4
	Replicate 3	Amplicon 55	A	MID8	ITS1F	B	MID11	ITS2
		Amplicon 56	A	MID8	ITS3	B	MID11	ITS4
		Amplicon 57	A	MID8	ITS86F	B	MID11	ITS4
	Replicate 4	Amplicon 58	A	MID11	ITS1F	B	MID8	ITS2
		Amplicon 59	A	MID11	ITS3	B	MID8	ITS4
		Amplicon 60	A	MID11	ITS86F	B	MID8	ITS4

Sample 6	Replicate 1	Amplicon 61	A	MID9	ITS1F	B	MID11	ITS2
		Amplicon 62	A	MID9	ITS3	B	MID11	ITS4
		Amplicon 63	A	MID9	ITS86F	B	MID11	ITS4
	Replicate 2	Amplicon 64	A	MID11	ITS1F	B	MID9	ITS2
		Amplicon 65	A	MID11	ITS3	B	MID9	ITS4
		Amplicon 66	A	MID11	ITS86F	B	MID9	ITS4
	Replicate 3	Amplicon 67	A	MID10	ITS1F	B	MID11	ITS2
		Amplicon 68	A	MID10	ITS3	B	MID11	ITS4
		Amplicon 69	A	MID10	ITS86F	B	MID11	ITS4
	Replicate 4	Amplicon 70	A	MID11	ITS1F	B	MID10	ITS2
		Amplicon 71	A	MID11	ITS3	B	MID10	ITS4
		Amplicon 72	A	MID11	ITS86F	B	MID10	ITS4
Sample 7	Replicate 1	Amplicon 73	A	MID11	ITS1F	B	MID11	ITS2
		Amplicon 74	A	MID11	ITS3	B	MID11	ITS4
		Amplicon 75	A	MID11	ITS86F	B	MID11	ITS4
	Replicate 2	Amplicon 76	A	MID12	ITS1F	B	MID12	ITS2
		Amplicon 77	A	MID12	ITS3	B	MID12	ITS4
		Amplicon 78	A	MID12	ITS86F	B	MID12	ITS4
	Replicate 3	Amplicon 79	A	MID12	ITS1F	B	MID7	ITS2
		Amplicon 80	A	MID12	ITS3	B	MID7	ITS4
		Amplicon 81	A	MID12	ITS86F	B	MID7	ITS4
	Replicate 4	Amplicon 82	A	MID8	ITS1F	B	MID12	ITS2
		Amplicon 83	A	MID8	ITS3	B	MID12	ITS4
		Amplicon 84	A	MID8	ITS86F	B	MID12	ITS4

Sequence name*	Primer sequence (5'-3')	Reference
A	CGTATCGCCTCCCTCGCGCCATCAG	
B	CTATGCGCCTTGCCAGCCCGCTCAG	
ITS1F	CTTGGTCATTTAGAGGAAGTAA	[19]
ITS2	GCTGCGTTCTTCATCGATGC	[18]
ITS3	GCATCGATGAAGAACGCAGC	[18]
ITS4	TCCTCCGCTTATTGATATGC	[18]
ITS86F	GTGAATCATCGAATCTTTGAA	[26]
MID7	CGTGTCTCTA	
MID8	CTCGCGTGTC	
MID9	TAGTATCAGC	
MID10	TCTCTATGCG	
MID11	TGATACGTCT	
MID12	TACTGAGCTA	

* Abbreviations used

A: 454 pyrosequencing adapter A

B: 454 pyrosequencing adapter B

MID: Multiplex identifier

ITS: Internal transcribed spacer
