# Supplementary material for: Comparison and Validation of Some ITS Primer Pairs Useful for Fungal Metabarcoding Studies
Source: PLoS One. 2014 Jun 16;9(6):e97629. doi: 10.1371/journal.pone.0097629 (PMC4059633; doi:10.1371/journal.pone.0097629)
Supplement: Table S3 — Complete list of OTUs identified in the 454 amplicon pyrosequencing experiment and corresponding BLAST scores and E-values. (PDF) [file pone.0097629.s005.pdf]

Supporting Information Table S3

The following table shows the selected BLAST hit References, E-values and corresponding BLAST scores for all OTUs identified at a 97% sequence similarity cut-off in the current study. OTUs were blasted against the UNITE and INSD databases using the massBLASTer tool available from PlutoF v2.0 (http://unite.ut.ee/workbench.php). OTUs that did not have a BLAST hit in the UNITE or INSD databases are indicated as “not applicable (NA)”

Reference	Score	E-value
Abrothallus suecicus	318	5.E-86
Absconditella lignicola	163	3.E-39
Absconditella lignicola	168	1.E-40
Absidia caerulea	459	2.E-128
Absidia caerulea	470	1.E-131
Acanthostigma perpusillum	311	8.E-84
Acephala macrosclerotiorum	343	1.E-93
Acephala macrosclerotiorum	388	6.E-107
Acephala macrosclerotiorum	436	1.E-121
Acremonium sp BCC 14080	340	1.E-92
Acremonium strictum	411	1.E-113
Acremonium strictum	315	7.E-85
Agaricus pinsitus	542	4.E-153
Agaricus pinsitus	529	2.E-149
Alatospora acuminata	180	3.E-44
Aliquandostipite khaoyaiensis	87.2	2.E-16
Alternaria alternata	65	2.E-09
Alternaria alternata	412	2.E-114
Alternaria consortialis	481	5.E-135
Alternaria rosae	484	5.E-136
Alternaria tenuissima	486	2.E-136
Alternaria triticina	115	6.E-25
Alternaria triticina	462	2.E-129
Amanita muscaria	586	2.E-166
Anhellia nectandrae	355	7.E-97
Antarctic yeast CBS 8941	473	1.E-132
Antarctic yeast CBS 8941	425	4.E-118
Anthracothecium prasinum	87.9	2.E-16
Anthracothecium prasinum	87.9	2.E-16
Arachnopeziza aurata	396	3.E-109
Arachnopeziza aurata	348	6.E-95
Arnium gigantosporum	288	5.E-77
Arnium macrotheca	467	9.E-131
Arnium macrotheca	433	2.E-120

Arthrinium sacchari	477	1.E-133
Arthrinium sacchari	475	3.E-133
Arthrobotrys oligospora	85.6	1.E-15
Articulospora proliferata	431	8.E-120
Articulospora proliferata	390	1.E-107
Ascobrunneispora aquatica	229	4.E-59
Ascomycota sp 6 RB 2011	383	1.E-105
Ascomycota sp 6 RB 2011	340	1.E-92
Ascomycota sp AM12	374	2.E-102
Ascomycota sp AR 2010	420	2.E-116
Ascomycota sp AR 2010	416	2.E-115
Ascomycota sp AR 2010	370	1.E-101
Ascomycota sp ARIZ OCAsh3 11	393	2.E-108
Ascomycota sp ARIZ OCAsh3 11	335	5.E-91
Ascomycota sp CH Co12	457	1.E-127
Ascomycota sp GMU LL 02 B3	443	1.E-123
Ascomycota sp GMU LL 02 B3	412	2.E-114
Ascomycota sp I306	391	6.E-108
Ascomycota sp r433	150	2.E-35
Aspergillus viridinutans	497	8.E-140
Aspicilia cinerea	252	7.E-66
Aspicilia dendroplaca	265	6.E-70
Aspicilia verruculosa	320	2.E-86
Asteridiella obesa	74.5	2.E-12
Asterophora sp aurim714	332	3.E-90
Athelia epiphylla	420	2.E-116
Athelopsis lembospora	484	6.E-136
Aulographina pinorum	361	8.E-99
Aureobasidium pullulans	405	3.E-112
Aureobasidium pullulans	480	1.E-134
Aureobasidium pullulans	457	1.E-127
Aureobasidium pullulans	195	8.E-49
Aureobasidium pullulans	187	1.E-46
Bacidina chloroticula	392	3.E-108
Bacidina flavoleprosa	142	5.E-33
Bacidina flavoleprosa	114	2.E-24
Barriopsis fusca	237	1.E-61
Basidiodendron caesiocinereum	163	4.E-39
Basidiodendron caesiocinereum	100	2.E-20
Beauveria pseudobassiana	468	5.E-131
Bellemerea alpina	196	2.E-49
Belonopsis eriophori	359	3.E-98
Betamyces americaemeridionalis	87.2	2.E-16
Betamyces americaemeridionalis	193	5.E-48
Betamyces americaemeridionalis	86.1	7.E-16
Betamyces americaemeridionalis	86.1	7.E-16
Bipolaris sorokiniana	391	6.E-108

Bjerkandera fumosa	510	9.E-144
Boletus edulis	448	4.E-125
Boletus edulis	732	0.E+00
Botryobambusa fusicoccum	375	3.E-103
Botryosporium longibrachiatum	97.1	3.E-19
Botryotinia fuckeliana	462	2.E-129
Botryotinia fuckeliana	440	1.E-122
Bovista dermoxantha	547	7.E-155
Brunneodinemasporium brasiliense	337	1.E-91
Brunneodinemasporium brasiliense	272	4.E-72
Bryoglossum gracile	348	4.E-95
Bullera globispora	245	5.E-64
Bullera sakaeratica	226	3.E-58
Cadophora finlandica	454	6.E-127
Cadophora finlandica	435	4.E-121
Cadophora finlandica	296	2.E-79
Cadophora finlandica	339	4.E-92
Calcarisporiella thermophila	161	2.E-38
Calcarisporiella thermophila	67.6	2.E-10
Calcarisporiella thermophila	67.6	2.E-10
Calcarisporiella thermophila	67.6	2.E-10
Calcarisporiella thermophila	67.6	2.E-10
Calcarisporium arbuscula	336	4.E-91
Calyptrozyma arxii	324	6.E-88
Calyptrozyma arxii	351	5.E-96
Camarographium koreanum	320	2.E-86
Camarographium koreanum	276	3.E-73
Candida homilentoma	85.6	8.E-16
Candida novakii	240	2.E-62
Candida novakii	171	2.E-41
Candida ontarioensis	103	6.E-21
Candida santamariae	527	7.E-149
Candida santamariae	510	9.E-144
Capronia pulcherrima	337	1.E-91
Capronia sp 94003b	331	6.E-90
Capronia sp 94003b	407	1.E-112
Capronia sp 94003b	420	1.E-116
Capronia sp 94003b	381	6.E-105
Capronia sp 94003b	387	1.E-106
Capronia sp 94003b	320	1.E-86
Capronia sp 94003b	309	2.E-83
Capronia sp 94003b	320	1.E-86
Capronia sp 94006a	288	8.E-77
Capronia sp 96003a	323	2.E-87
Capronia sp 96003a	340	1.E-92
Capronia sp 96003a	366	2.E-100
Capronia sp 96003a	302	4.E-81

Capronia sp 96003a	322	3.E-87
Catenulifera brachyconia	426	2.E-118
Catenulifera brevicollaris	329	2.E-89
Catenulostroma hermanusense	364	7.E-100
Catenulostroma hermanusense	444	8.E-124
Catenulostroma microsporum	465	3.E-130
Catenulostroma protearum	374	1.E-102
Catenulostroma protearum	391	7.E-108
Catenulostroma protearum	372	3.E-102
Catenulostroma protearum	363	2.E-99
Cenococcum geophilum	431	8.E-120
Cenococcum geophilum	399	2.E-110
Ceratocystis paradoxa	480	1.E-134
Ceratocystis paradoxa	457	1.E-127
Cercophora sulphurella	168	1.E-40
Cercophora sulphurella	137	1.E-31
Cercospora sophorae	551	5.E-156
Chaenothecopsis pusiola	65	1.E-09
Chaetomidium arxii	453	1.E-126
Chaetomium aureum	486	2.E-136
Chaetomium aureum	464	6.E-130
Chaetomium aureum	207	1.E-52
Chaetomium cupreum	431	6.E-120
Chaetomium jodhpurense	370	1.E-101
Chaetomium nigricolor	473	1.E-132
Chaetosphaeria bombycina	253	2.E-66
Chaetosphaeria dilabens	331	9.E-90
Chaetothyriales sp 16708	484	5.E-136
Chaetothyriales sp 16708	457	1.E-127
Chalara microspora	462	2.E-129
Chalara microspora	401	5.E-111
Chalara microspora	298	6.E-80
Chalara pseudoaffinis	410	1.E-113
Chlamydotubeufia khunkornensis	180	2.E-44
Chlorociboria argentinensis	215	4.E-55
Chrysosporium merdarium	415	5.E-115
Chrysosporium pseudomerdarium	401	5.E-111
Cistella acuum	294	8.E-79
Cistella grevillei	391	7.E-108
Cistella grevillei	355	4.E-97
Cistella spicicola	344	8.E-94
Cladonia borealis	446	2.E-124
Cladonia coniocraea	63.4	6.E-09
Cladonia diversa	407	1.E-112
Cladonia fimbriata	142	7.E-33
Cladonia fimbriata	136	6.E-31
Cladonia fimbriata	136	6.E-31

Cladonia fimbriata	137	2.E-31
Cladonia fimbriata	148	9.E-35
Cladonia fimbriata	153	3.E-36
Cladonia fimbriata	458	8.E-128
Cladonia fimbriata	136	6.E-31
Cladonia fimbriata	114	3.E-24
Cladonia fimbriata	117	4.E-25
Cladonia fimbriata	117	3.E-25
Cladonia fimbriata	148	9.E-35
Cladonia fimbriata	148	9.E-35
Cladonia fimbriata	150	3.E-35
Cladonia fimbriata	136	6.E-31
Cladonia fimbriata	139	7.E-32
Cladonia fimbriata	134	2.E-30
Cladonia fimbriata	145	8.E-34
Cladonia fimbriata	110	3.E-23
Cladonia fimbriata	125	1.E-27
Cladonia fimbriata	145	8.E-34
Cladonia fimbriata	137	1.E-31
Cladonia fimbriata	120	4.E-26
Cladonia fimbriata	137	2.E-31
Cladonia fimbriata	141	2.E-32
Cladonia fimbriata	129	5.E-29
Cladonia fimbriata	134	2.E-30
Cladonia fimbriata	139	7.E-32
Cladonia fimbriata	286	2.E-76
Cladonia fimbriata	136	7.E-31
Cladonia fimbriata	131	1.E-29
Cladonia fimbriata	150	3.E-35
Cladonia fimbriata	156	4.E-37
Cladonia fimbriata	117	4.E-25
Cladonia fimbriata	141	2.E-32
Cladonia fimbriata	423	2.E-117
Cladonia fimbriata	117	3.E-25
Cladonia fimbriata	136	6.E-31
Cladonia fimbriata	142	8.E-33
Cladonia fimbriata	117	4.E-25
Cladonia fimbriata	129	5.E-29
Cladonia fimbriata	115	1.E-24
Cladonia fimbriata	114	3.E-24
Cladonia fimbriata	131	2.E-29
Cladonia fimbriata	132	1.E-29
Cladonia fimbriata	427	9.E-119
Cladonia fimbriata	416	2.E-115
Cladonia foliacea	416	2.E-115
Cladonia furcata	350	1.E-95
Cladonia gracilis	443	1.E-123

Cladonia gracilis subsp elongata	448	5.E-125
Cladonia grayi	491	7.E-138
Cladonia grayi	347	2.E-94
Cladonia grayi	466	2.E-130
Cladonia merochlorophaea	340	1.E-92
Cladophialophora chaetospira	391	6.E-108
Cladophialophora chaetospira	114	2.E-24
Cladophialophora chaetospira	253	2.E-66
Cladophialophora chaetospira	345	5.E-94
Cladophialophora humicolae	549	2.E-155
Cladophialophora humicolae	665	0.E+00
Cladophialophora minutissima	383	1.E-105
Cladophialophora minutissima	318	5.E-86
Cladophialophora minutissima	331	8.E-90
Cladophialophora minutissima	331	8.E-90
Cladophialophora minutissima	348	4.E-95
Cladophialophora minutissima	394	8.E-109
Cladophialophora minutissima	418	6.E-116
Cladophialophora minutissima	413	2.E-114
Cladophialophora minutissima	394	7.E-109
Cladophialophora minutissima	355	7.E-97
Cladophialophora minutissima	440	1.E-122
Cladophialophora minutissima	442	3.E-123
Cladophialophora minutissima	357	1.E-97
Cladophialophora minutissima	398	6.E-110
Cladophialophora minutissima	374	1.E-102
Cladophialophora minutissima	392	3.E-108
Cladophialophora minutissima	344	8.E-94
Cladophialophora modesta	207	1.E-52
Cladophialophora scillae	285	6.E-76
Cladosporium oxysporum	367	8.E-101
Claroideoglomus claroideum	56.5	5.E-07
Claroideoglomus claroideum	56.5	5.E-07
Claroideoglomus claroideum	56.5	5.E-07
Claroideoglomus claroideum	56.5	5.E-07
Claroideoglomus claroideum	56.5	6.E-07
Claroideoglomus claroideum	56.5	5.E-07
Claroideoglomus claroideum	56.5	5.E-07
Claviradulomyces dabeicola	237	2.E-61
Claviradulomyces dabeicola	182	8.E-45
Clitocybe vermicularis	223	3.E-57
Clitopilus hobsonii	434	1.E-120
Clonostachys rosea	497	8.E-140
Clonostachys rosea	477	8.E-134
Coccomyces mucronatus	131	8.E-30
Coemansia asiatica	58.4	1.E-07
Coemansia asiatica	58.4	1.E-07

Coemansia asiatica	56.5	5.E-07
Coleophoma eucalyptorum	362	3.E-99
Coleophoma eucalyptorum	364	9.E-100
Collophora hispanica	386	2.E-106
Collophora hispanica	342	4.E-93
Collophora hispanica	296	2.E-79
Collophora hispanica	346	2.E-94
Collophora hispanica	241	1.E-62
Collophora paarla	388	6.E-107
Coniochaeta gigantospora	442	4.E-123
Coniochaeta gigantospora	401	5.E-111
Coniochaeta prunicola	169	5.E-41
Coniothyrium fuckelii	465	3.E-130
Coniothyrium fuckelii	429	3.E-119
Coniothyrium fuckelii	433	2.E-120
Conlarium duplumascospora	390	1.E-107
Conocybe echinata	545	3.E-154
Coprinellus disseminatus	559	2.E-158
Coprinellus disseminatus	553	2.E-156
Coprinellus verrucispermus	523	1.E-147
Coprinopsis atramentaria	546	1.E-154
Coprinopsis cinerea	545	4.E-154
Cordana pauciseptata	291	1.E-77
Cordyceps memorabilis	380	1.E-104
Cortinarius odorifer	168	2.E-40
Cortinarius parvannulatus	516	2.E-145
Corynascus kuwaitiensis	516	2.E-145
Corynascus kuwaitiensis	416	2.E-115
Cosmospora vilior	245	4.E-64
Cryptococcus	324	6.E-88
Cryptococcus aerius	559	2.E-158
Cryptococcus aerius	545	3.E-154
Cryptococcus aff amylolyticus AS 22398	484	6.E-136
Cryptococcus aff amylolyticus AS 22398	381	6.E-105
Cryptococcus aff amylolyticus AS 22398	141	1.E-32
Cryptococcus aff amylolyticus AS 22398	265	7.E-70
Cryptococcus aff amylolyticus AS 22398	318	6.E-86
Cryptococcus aff amylolyticus AS 22398	379	2.E-104
Cryptococcus aff amylolyticus AS 22398	462	2.E-129
Cryptococcus aff laurentii D 0721a1	191	1.E-47
Cryptococcus cylindricus	560	1.E-158
Cryptococcus dimennae	204	2.E-51
Cryptococcus dimennae	126	3.E-28
Cryptococcus dimennae	130	2.E-29
Cryptococcus flavus	198	7.E-50
Cryptococcus laurentii	215	7.E-55
Cryptococcus laurentii	147	3.E-34

Cryptococcus laurentii	492	3.E-138
Cryptococcus paraflavus	258	9.E-68
Cryptococcus podzolicus	489	2.E-137
Cryptococcus podzolicus	269	4.E-71
Cryptococcus podzolicus	468	5.E-131
Cryptococcus podzolicus	200	2.E-50
Cryptococcus podzolicus	111	9.E-24
Cryptococcus podzolicus	130	2.E-29
Cryptococcus podzolicus	202	6.E-51
Cryptococcus podzolicus	231	7.E-60
Cryptococcus podzolicus	119	5.E-26
Cryptococcus randhawii	578	4.E-164
Cryptococcus randhawii	568	6.E-161
Cryptococcus sp BI20	196	2.E-49
Cryptococcus sp VPCI 1367 B1	111	8.E-24
Cryptococcus terricola	380	1.E-104
Cryptococcus terricola	458	6.E-128
Cryptococcus terricola	604	5.E-172
Cryptococcus terricola	235	7.E-61
Cryptococcus victoriae	329	2.E-89
Cryptococcus victoriae	404	9.E-112
Cryptococcus victoriae	416	2.E-115
Cryptococcus wieringae	599	2.E-170
Cryptosporiopsis actinidiae	104	2.E-21
Cryptosporiopsis actinidiae	380	2.E-104
Cryptosporiopsis actinidiae	169	5.E-41
Cudoniella acicularis	271	1.E-71
Cudoniella acicularis	399	3.E-110
Cudoniella clavus	378	4.E-104
Curreya pityophila	369	3.E-101
Cyphellophora hylomeconis	385	6.E-106
Dactylaria lanosa	340	1.E-92
Dactylella oviparasitica	163	4.E-39
Dactylellina drechsleri	504	1.E-141
Dactylellina drechsleri	462	2.E-129
Dactylellina ellipsospora	478	4.E-134
Dactylellina phymatopaga	174	1.E-42
Daldinia fissa	396	2.E-109
Davidiella tassiana	436	1.E-121
Debaryomyces hansenii	529	2.E-149
Debaryomyces polymorphus var polymorphus	543	1.E-153
Debaryomyces polymorphus var polymorphus	525	3.E-148
Degelia gayana	313	2.E-84
Degelia gayana	213	2.E-54
Devriesia pseudoamericana	350	2.E-95
Devriesia pseudoamericana	369	3.E-101
Devriesia pseudoamericana	298	6.E-80

Devriesia pseudoamericana	296	2.E-79
Devriesia pseudoamericana	281	6.E-75
Devriesia pseudoamericana	335	5.E-91
Dinemasporium morbidum	356	2.E-97
Dinemasporium morbidum	480	1.E-134
Dinemasporium pseudostrigosum	439	4.E-122
Dinemasporium pseudostrigosum	414	6.E-115
Dinemasporium pseudostrigosum	363	2.E-99
Dinemasporium strigosum	266	3.E-70
Dinemasporium strigosum	400	8.E-111
Dinemasporium strigosum	374	8.E-103
Dioszegia athyri	425	3.E-118
Dioszegia rishiriensis	415	4.E-115
Dokmaia monthadangii	459	2.E-128
Dothideomycetes sp DC2167	448	6.E-125
Dothideomycetes sp genotype 188	313	2.E-84
Dothideomycetes sp genotype 188	259	3.E-68
Drechslera erythrospila	415	5.E-115
Drechslera erythrospila	475	4.E-133
Drechslera erythrospila	460	8.E-129
Drechslera nobleae	304	1.E-81
Drechslera nobleae	329	3.E-89
Drechslera poae	381	6.E-105
Elsinoe ampelina	320	2.E-86
Emericella nidulans	123	4.E-27
Emericella nidulans	109	8.E-23
Emericella nidulans	155	1.E-36
Emericella nidulans	381	6.E-105
Emericella nidulans	147	3.E-34
Emericella purpurea	125	7.E-28
Emmonsia parva	519	2.E-146
Entoloma conferendum	439	4.E-122
Epacris microphylla root associated fungus 12	453	2.E-126
Epacris microphylla root associated fungus 12	435	5.E-121
Epacris microphylla root associated fungus 17	472	3.E-132
Epacris microphylla root associated fungus 21	431	8.E-120
Epacris microphylla root associated fungus 21	313	1.E-84
Epacris microphylla root associated fungus 21	315	5.E-85
Epacris microphylla root associated fungus 26	321	5.E-87
Epacris microphylla root associated fungus 26	425	3.E-118
Epacris microphylla root associated fungus 26	337	1.E-91
Epacris microphylla root associated fungus 26	331	6.E-90
Epacris microphylla root associated fungus 26	327	7.E-89
Epacris microphylla root associated fungus 33	439	3.E-122
Epacris microphylla root associated fungus 33	402	3.E-111
Epacris microphylla root associated fungus 33	488	4.E-137
Epacris microphylla root associated fungus 33	425	3.E-118

Epacris microphylla root associated fungus 33	346	2.E-94
Epacris pulchella root associated fungus EP20	427	9.E-119
Epacris pulchella root associated fungus EP20	490	1.E-137
Epacris pulchella root associated fungus EP26	167	2.E-40
Epacris pulchella root associated fungus EP54	448	5.E-125
Epacris pulchella root associated fungus EP55	401	5.E-111
Epicoccum nigrum	347	1.E-94
Eudarluca caricis	212	5.E-54
Exobasidium kishianum	455	4.E-127
Exobasidium rostrupii	443	1.E-123
Exophiala bergeri	418	5.E-116
Exophiala equina	215	6.E-55
Exophiala eucalyptorum	491	7.E-138
Exophiala placitae	389	2.E-107
Exophiala sideris	462	3.E-129
Exophiala sideris	442	3.E-123
Filobasidium uniguttulatum	331	6.E-90
Filobasidium uniguttulatum	622	2.E-177
Friedmanniomyces endolithicus	367	1.E-100
Friedmanniomyces endolithicus	311	8.E-84
fungal endophyte	340	1.E-92
fungal sp 2747 YZ 2011	416	2.E-115
fungal sp ARIZ AZ0780	433	2.E-120
fungal sp NLEndoHerit 014 2008N5 26 2N	327	8.E-89
Funneliformis geosporum	109	6.E-23
Funneliformis geosporum	112	7.E-24
Funneliformis geosporum	109	7.E-23
Funneliformis geosporum	112	8.E-24
Fusarium biseptatum	355	7.E-97
Fusarium culmorum	355	5.E-97
Fusarium culmorum	336	3.E-91
Fusarium equiseti	322	5.E-87
Fusarium merismoides	457	1.E-127
Fusarium oxysporum	355	5.E-97
Fusarium oxysporum	391	6.E-108
Fusarium oxysporum	388	6.E-107
Fusarium oxysporum	448	6.E-125
Fusarium torulosum	497	8.E-140
Fusarium torulosum	466	2.E-130
Fusicladium cordae	502	3.E-141
Fusicladium cordae	277	2.E-73
Fusicladium cordae	484	5.E-136
Fusicladium cordae	213	2.E-54
Fusicladium phillyreae	213	2.E-54
Gaertneriomyces semiglobifer	80.5	4.E-14
Gammamyces ourimbahensis	85.6	6.E-16
Ganoderma adspersum	538	4.E-152

Ganoderma applanatum	440	1.E-122
Ganoderma applanatum	542	4.E-153
Ganoderma applanatum	529	2.E-149
Geoglossum atropurpureum	174	1.E-42
Geomyces pannorum	462	2.E-129
Geomyces pannorum	393	2.E-108
Geomyces vinaceus	442	3.E-123
Geopora clausa	82.4	6.E-15
Geosmithia langdonii	183	2.E-45
Geosmithia putterillii	137	2.E-31
Glomerella graminicola	453	1.E-126
Glomus custos	126	4.E-28
Glomus intraradices	106	6.E-22
Glomus sp 3 SUN 2011	95.1	9.E-19
Guignardia citricarpa	95.3	8.E-19
Helicodendron multiseptatum	440	1.E-122
Helotiaceae sp IV GK 2010	320	1.E-86
Helotiales sp 1 CG 2012	312	4.E-84
Helotiales sp 1 MV 2011	432	3.E-120
Helotiales sp 1 MV 2011	387	1.E-106
Helotiales sp 16 MV 2011	434	9.E-121
Helotiales sp 16 MV 2011	431	8.E-120
Helotiales sp 16 MV 2011	383	2.E-105
Helotiales sp 16 MV 2011	348	6.E-95
Helotiales sp 2 BB 2010	378	4.E-104
Helotiales sp 27 MV 2011	427	8.E-119
Helotiales sp 27 MV 2011	444	8.E-124
Helotiales sp 5 CG 2012	318	4.E-86
Helotiales sp I12F 02299	305	4.E-82
Helotiales sp MU 2009 3	345	4.E-94
Helotiales sp ODKB3	440	1.E-122
Helotiales sp PIMO 265	247	1.E-64
Helotiales sp REF045	405	3.E-112
Helotiales sp REF055	315	4.E-85
Helotiales sp SC3 4	467	9.E-131
Helotiales sp SC9 3	388	5.E-107
Helotiales sp SL11101	233	2.E-60
Helotiales sp WMM 2012b	443	1.E-123
Helotiales sp WMM 2012b	407	1.E-112
Helotiales sp WMM 2012g	336	3.E-91
Helotiales sp WMM 2012g	320	2.E-86
Helvella maculata	291	8.E-78
Helvella maculata	264	1.E-69
Herpotrichiellaceae sp RB 2011	337	1.E-91
Heterobasidion annosum	415	4.E-115
Hirsutella minnesotensis	497	8.E-140
Homortomyces combreti	215	6.E-55

Humicolopsis cephalosporioides	404	1.E-111
Humicolopsis cephalosporioides	361	8.E-99
Humicolopsis cephalosporioides	272	4.E-72
Humicolopsis cephalosporioides	316	2.E-85
Hyaloscypha hepaticola	385	4.E-106
Hyaloscypha sp 2 13c	366	3.E-100
Hydnotrya tulasnei	551	5.E-156
Hydnotrya tulasnei	538	4.E-152
Hyphodontia breviseta	385	6.E-106
Hypholoma fasciculare	499	3.E-140
Hypholoma fasciculare	567	9.E-161
Hypocrea lixii	510	1.E-143
Hypocrea lixii	492	3.E-138
Hypocrea voglmayrii	259	3.E-68
Hypogymnia inactiva	148	6.E-35
Hypomyces cervinigenus	326	3.E-88
Infundichalara microchona	405	4.E-112
Infundichalara microchona	350	1.E-95
Infundichalara microchona	369	3.E-101
Infundichalara microchona	350	2.E-95
Infundichalara microchona	366	2.E-100
Inocybe lacera var lacera	480	1.E-134
Inocybe lacera var lacera	559	2.E-158
Inocybe lacera var lacera	545	3.E-154
Inocybe ochroalba	320	2.E-86
Inocybe ochroalba	233	2.E-60
Johansonia chapadiensis	150	3.E-35
Knufia chersonesos	247	2.E-64
Knufia chersonesos	449	2.E-125
Knufia chersonesos	180	3.E-44
Kockovaella schimae	383	2.E-105
Kurtzmanomyces nectairei	215	8.E-55
Lachnum brevipilosum	301	7.E-81
Lachnum sp 1 MV 2011	440	1.E-122
Lachnum sp 1 MV 2011	453	2.E-126
Lachnum sp 1 MV 2011	420	1.E-116
Lachnum sp 1 MV 2011	409	3.E-113
Lachnum sp 252	318	5.E-86
Lachnum virgineum	150	2.E-35
Lasiosphaeria ovina	248	6.E-65
Lecanicillium psalliotae	374	9.E-103
Lecanicillium psalliotae	492	2.E-138
Lecanicillium psalliotae	472	4.E-132
Lecanicillium psalliotae	329	2.E-89
Lecythophora sp BESC803p	446	2.E-124
Lecythophora sp YP363	271	1.E-71
Lenzites betulinus	556	2.E-157

Leotiomycetes sp ASR H18 12A	301	9.E-81
Leotiomycetes sp ASR H18 12A	259	2.E-68
Leotiomycetes sp ASR H18 12A	307	1.E-82
Leotiomycetes sp F21	321	7.E-87
Leotiomycetes sp genotype 134	364	6.E-100
Leotiomycetes sp NK264	462	2.E-129
Leotiomycetes sp NK264	366	3.E-100
Leotiomycetes sp NK264	431	6.E-120
Leotiomycetes sp NK266	420	2.E-116
Lepraria aff obtusatica BRY C56005	93.5	3.E-18
Lepraria elobata	228	9.E-59
Leptosphaeria doliolum	296	3.E-79
Leptosphaeria korrae	351	5.E-96
Leptosphaerulina chartarum	465	3.E-130
Leptosphaerulina chartarum	455	4.E-127
Leuconeurospora sp T11Cd2	377	1.E-103
Limnoperdon incarnatum	257	1.E-67
Linderina macrospora	71.3	2.E-11
Lobaria retigera	241	1.E-62
Lobaria retigera	195	8.E-49
Lobariella pallida	267	1.E-70
Lophiostoma chamaecyparidis	374	1.E-102
Lophiostoma chamaecyparidis	372	4.E-102
Lophiostoma chamaecyparidis	318	5.E-86
Lophiostoma chamaecyparidis	344	8.E-94
Lophiostoma cynaroidis	294	8.E-79
Lophodermium baculiferum	78.7	8.E-14
Lophodermium conigenum	125	9.E-28
Lophodermium conigenum	248	5.E-65
Lophodermium pinastri	464	8.E-130
Lophodermium pinastri	464	8.E-130
Lophodermium pinastri	438	3.E-122
Lophodermium pinastri	438	3.E-122
Lophodermium seditiosum	451	5.E-126
Lycoperdon aff pyriforme Scl 6 1 1L	553	2.E-156
Lyophyllum sp Cultivar Jpn	56.5	6.E-07
Macroconia leptosphaeriae	250	1.E-65
Macroconia leptosphaeriae	189	3.E-47
Magnaporthe poae	147	3.E-34
Malassezia globosa	462	3.E-129
Malassezia globosa	458	7.E-128
Malassezia globosa	554	6.E-157
Malassezia globosa	579	3.E-164
Malassezia restricta	540	1.E-152
Malassezia restricta	456	2.E-127
Malassezia restricta	670	0.E+00
Malassezia restricta	678	0.E+00

Malassezia sympodialis	110	1.E-23
Malassezia sympodialis	651	0.E+00
Massariosphaeria typhicola	536	2.E-151
Melanelixia piliferella	168	1.E-40
Melanocarpus albomyces	386	2.E-106
Melanotaenium euphorbiae	103	6.E-21
Melanotaenium euphorbiae	103	6.E-21
Meliniomyces bicolor	459	2.E-128
Meliniomyces bicolor	307	1.E-82
Meliniomyces bicolor	335	5.E-91
Meliniomyces bicolor	374	1.E-102
Meliniomyces sp GK 2010	183	2.E-45
Meliniomyces sp SM7 2	392	3.E-108
Meliniomyces variabilis	383	2.E-105
Meliniomyces vraolstadiae	351	5.E-96
Metarhizium flavoviride	340	1.E-92
Metschnikowia hawaiiensis	87.2	2.E-16
Metschnikowia hawaiiensis	87.2	2.E-16
Metschnikowia hawaiiensis	87.2	2.E-16
Metschnikowia hawaiiensis	87.2	2.E-16
Metschnikowia pulcherrima	88.8	8.E-17
Micarea denigrata	296	2.E-79
Micarea hedlundii	225	8.E-58
Micarea hedlundii	167	2.E-40
Micarea hedlundii	161	8.E-39
Microdochium bolleyi	391	6.E-108
Microdochium bolleyi	379	2.E-104
Microscypha ellisii	375	3.E-103
Microsphaeropsis arundinis	459	2.E-128
Microsphaeropsis arundinis	424	1.E-117
Microsphaeropsis arundinis	418	5.E-116
Mollisia incrustata	237	1.E-61
Monoblepharis hypogyna	183	2.E-45
Mortierella alpina	285	5.E-76
Mortierella alpina	182	6.E-45
Mortierella alpina	573	1.E-162
Mortierella angusta	617	6.E-176
Mortierella angusta	514	8.E-145
Mortierella bainieri	381	1.E-104
Mortierella bainieri	276	5.E-73
Mortierella cystojenkinii	603	2.E-171
Mortierella elongata	353	1.E-96
Mortierella elongata	427	7.E-119
Mortierella elongata	636	0.E+00
Mortierella jenkinii	141	2.E-32
Mortierella lignicola	272	3.E-72
Mortierella lignicola	605	4.E-172

Mortierella lignicola	612	3.E-174
Mortierella macrocystis	595	3.E-169
Mortierella parvispora	537	1.E-151
Mortierella parvispora	616	2.E-175
Mortierella polycephala	76.8	5.E-13
Mortierella turficola	436	2.E-121
Mortierellaceae sp PDKB9	610	1.E-173
Mortierellaceae sp PDKB9	612	3.E-174
Mucor moelleri	122	1.E-26
Mycena arcangeliana	569	3.E-161
Mycena arcangeliana	555	4.E-157
Mycena corynephora	154	2.E-36
Mycena epipterygia	562	3.E-159
Mycena galopus	569	3.E-161
Mycena galopus	560	9.E-159
Mycena maurella	93.5	4.E-18
Mycena meliigena	423	3.E-117
Mycena metata	416	2.E-115
Mycena simia	547	7.E-155
Mycoblastus sanguinarioides	202	4.E-51
Myrmecridium banksiae	336	3.E-91
Myrmecridium banksiae	402	3.E-111
Myrmecridium banksiae	366	2.E-100
Myrmecridium phragmitis	274	1.E-72
Myrmecridium phragmitis	293	3.E-78
Myrmecridium phragmitis	198	6.E-50
Myrmecridium schulzeri	324	6.E-88
Myrmecridium schulzeri	445	4.E-124
Myrothecium gramineum	206	5.E-52
NA1	NA	NA
NA10	NA	NA
NA11	NA	NA
NA12	NA	NA
NA13	NA	NA
NA14	NA	NA
NA15	NA	NA
NA16	NA	NA
NA17	NA	NA
NA18	NA	NA
NA19	NA	NA
NA2	NA	NA
NA20	NA	NA
NA21	NA	NA
NA22	NA	NA
NA23	NA	NA
NA24	NA	NA
NA25	NA	NA

NA26	NA	NA
NA27	NA	NA
NA28	NA	NA
NA29	NA	NA
NA3	NA	NA
NA30	NA	NA
NA31	NA	NA
NA32	NA	NA
NA33	NA	NA
NA34	NA	NA
NA35	NA	NA
NA36	NA	NA
NA37	NA	NA
NA38	NA	NA
NA39	NA	NA
NA4	NA	NA
NA40	NA	NA
NA41	NA	NA
NA42	NA	NA
NA43	NA	NA
NA44	NA	NA
NA45	NA	NA
NA46	NA	NA
NA47	NA	NA
NA48	NA	NA
NA49	NA	NA
NA5	NA	NA
NA50	NA	NA
NA51	NA	NA
NA6	NA	NA
NA7	NA	NA
NA8	NA	NA
NA9	NA	NA
Naemacyclus niveus	464	8.E-130
Neocallimastix sp H GFM 2	96.7	4.E-19
Neocallimastix sp H GFM 2	95.1	1.E-18
Neocallimastix sp H GFM 2	95.1	9.E-19
Neocallimastix sp H GFM 2	95.1	9.E-19
Neocallimastix sp H GFM 2	77.7	2.E-13
Noosia banksiae	388	7.E-107
Ochrolechia juvenalis	153	2.E-36
Ogataea chonburiensis	126	3.E-28
Oidiodendron	NA	NA
Oidiodendron chlamydosporicum	439	3.E-122
Oidiodendron chlamydosporicum	410	1.E-113
Oidiodendron chlamydosporicum	364	6.E-100
Oidiodendron chlamydosporicum	403	1.E-111

Oidiodendron griseum	324	6.E-88
Oidiodendron griseum	280	2.E-74
Oidiodendron pilicola	435	3.E-121
Oidiodendron pilicola	392	3.E-108
Oidiodendron reticulatum	454	6.E-127
Oidiodendron reticulatum	425	3.E-118
Oidiodendron tenuissimum	97.1	2.E-19
Ophiocordyceps entomorrhiza	148	5.E-35
Ophiocordyceps irangiensis	63.4	4.E-09
Ophiocordyceps irangiensis	60.2	3.E-08
Ophiocordyceps nutans	95.1	8.E-19
Ophiocordyceps nutans	98.3	1.E-19
Ophiocordyceps prolifica	502	3.E-141
Ophiostoma bicolor	99.9	5.E-20
Paraconiothyrium brasiliense	145	7.E-34
Paraconiothyrium sporulosum	455	4.E-127
Paraphaeosphaeria michotii	383	1.E-105
Parastagonospora nodorum	355	6.E-97
Parastagonospora nodorum	361	7.E-99
Passalora zambiae	252	5.E-66
Penicillium aculeatum	457	1.E-127
Penicillium adametzii	494	7.E-139
Penicillium adametzii	473	1.E-132
Penicillium bialowiezense	423	2.E-117
Penicillium brevicompactum	228	9.E-59
Penicillium canescens	394	6.E-109
Penicillium canescens	480	1.E-134
Penicillium canescens	473	1.E-132
Penicillium cecidicola	364	8.E-100
Penicillium cecidicola	477	8.E-134
Penicillium citreonigrum	393	2.E-108
Penicillium concentricum	150	2.E-35
Penicillium corylophilum	483	2.E-135
Penicillium herquei	351	5.E-96
Penicillium janthinellum	412	4.E-114
Penicillium lanosum	492	2.E-138
Penicillium montanense	394	6.E-109
Penicillium montanense	483	2.E-135
Penicillium montanense	490	1.E-137
Penicillium montanense	460	8.E-129
Penicillium montanense	399	2.E-110
Penicillium namyslowskii	494	7.E-139
Penicillium namyslowskii	468	5.E-131
Penicillium ochrochloron	391	6.E-108
Penicillium ochrochloron	497	8.E-140
Penicillium raistrickii	403	1.E-111
Penicillium simile	422	4.E-117

Penicillium tularense	423	2.E-117
Penicillium tularense	394	8.E-109
Penidiella ellipsoidea	147	1.E-34
Peniophora lycii	444	9.E-124
Pesotum fragrans	82.4	5.E-15
Pezicula carpinea	258	8.E-68
Peziza ostracoderma	269	4.E-71
Peziza ostracoderma	448	7.E-125
Pezizella discreta	294	7.E-79
Pezizomycetes sp genotype 454	443	1.E-123
Phaeoacremonium griseorubrum	224	1.E-57
Phaeococcomyces catenatus	542	4.E-153
Phaeococcomyces catenatus	256	3.E-67
Phaeococcomyces chersonesos	201	1.E-50
Phaeococcomyces eucalypti	168	1.E-40
Phaeococcomyces eucalypti	307	1.E-82
Phaeococcomyces eucalypti	299	3.E-80
Phaeococcomyces nigricans	381	5.E-105
Phaeococcomyces nigricans	335	5.E-91
Phaeosphaeria avenaria	453	2.E-126
Phaeosphaeria avenaria	425	3.E-118
Phaeosphaeria herpotrichoides	449	2.E-125
Phaeosphaeria nodorum	451	5.E-126
Phaeosphaeria nodorum	449	2.E-125
Phaeosphaeria nodorum	405	4.E-112
Phaeosphaeria phragmitis	380	2.E-104
Phaeosphaeria phragmitis	414	6.E-115
Phaeosphaeria phragmitis	363	2.E-99
Phaeosphaeriopsis sp C652	453	2.E-126
Phaeosphaeriopsis sp TMS 2011	323	2.E-87
Phaeotheca fissurella	377	8.E-104
Phellinopsis conchata	183	2.E-45
Phellinus pomaceus	91.9	1.E-17
Phialemonium curvatum	266	3.E-70
Phialemonium curvatum	236	3.E-61
Phialemonium curvatum	275	5.E-73
Phialemonium curvatum	256	3.E-67
Phialemonium curvatum	247	2.E-64
Phialemonium curvatum	202	5.E-51
Phialocephala fortinii	393	2.E-108
Phialocephala fortinii	369	3.E-101
Phialocephala fortinii	436	1.E-121
Phialocephala fusca	396	2.E-109
Phialocephala virens	275	4.E-73
Phialocephala virens	337	1.E-91
Phialophora lignicola	336	3.E-91
Phialophora phaeophora	532	2.E-150

Phialophora sp DF36	446	1.E-124
Phialophora sp DF36	416	2.E-115
Phlebia livida subsp tuberculata	161	1.E-38
Phlyctochytrium africanum	195	1.E-48
Phlyctochytrium palustre	180	3.E-44
Pholiota populnea	573	1.E-162
Phoma betae	292	3.E-78
Phoma caloplacae	396	3.E-109
Phoma caloplacae	337	1.E-91
Phoma herbarum	294	8.E-79
Phoma macrostoma	320	1.E-86
Phyllachora phyllostachydis	345	5.E-94
Pichia kudriavzevii	57.1	3.E-07
Piriformospora sp X 30	90.3	3.E-17
Piriformospora sp X 30	90.3	3.E-17
Piriformospora sp X 30	90.3	3.E-17
Placynthiella icmalea	442	4.E-123
Placynthiella icmalea	401	5.E-111
Platismatia stenophylla	320	2.E-86
Plectania rhytidia	104	2.E-21
Pleopsidium chlorophanum	187	3.E-46
Pleospora herbarum	446	2.E-124
Pleosporales sp 28e	185	9.E-46
Pleosporales sp 28e	410	1.E-113
Pleosporales sp 28e	355	4.E-97
Pleosporales sp 5 TMS 2011	190	2.E-47
Pochonia bulbillosa	364	7.E-100
Pochonia bulbillosa	527	7.E-149
Pochonia bulbillosa	512	2.E-144
Pochonia suchlasporia	329	2.E-89
Podoscypha venustula	96.7	5.E-19
Podospora ellisiana	278	4.E-74
Podospora ellisiana	413	1.E-114
Podospora ellisiana	363	2.E-99
Podospora formosana	448	5.E-125
Podospora miniglutinans	420	1.E-116
Polyphlyctis unispina	161	1.E-38
Porosphaerella cordanophora	321	7.E-87
Porosphaerella cordanophora	263	3.E-69
Porosphaerella cordanophora	222	4.E-57
Powellomyces hirtus	79.2	7.E-14
Powellomyces hirtus	93.5	5.E-18
Powellomyces hirtus	82.4	1.E-14
Powellomyces hirtus	82.4	1.E-14
Preussia australis	470	1.E-131
Preussia dubia	415	5.E-115
Preussia minima	343	1.E-93

Pringsheimia euphorbiae	294	8.E-79
Pseudocercosporella fraxini	296	2.E-79
Pseudocercosporella fraxini	367	1.E-100
Pseudocercosporella fraxini	327	8.E-89
Pyrenochaetopsis microspora	435	3.E-121
Pyrenochaetopsis microspora	392	3.E-108
Pyrenophora tetrarrhenae	272	3.E-72
Pyrenula macrospora	115	7.E-25
Pyxine limbulata	99	7.E-20
Rachicladosporium pini	443	1.E-123
Rachicladosporium pini	420	1.E-116
Ramaria abietina	111	8.E-24
Ramichloridium strelitziae	252	5.E-66
Rhexocercosporidium panacis	461	7.E-129
Rhexocercosporidium panacis	431	6.E-120
Rhizophlyctis harderi	86.1	7.E-16
Rhizophlyctis rosea	320	2.E-86
Rhizophlyctis rosea	605	4.E-172
Rhizophlyctis rosea	172	6.E-42
Rhizophlyctis rosea	163	2.E-39
Rhizophlyctis rosea	562	3.E-159
Rhizophlyctis rosea	80.5	3.E-14
Rhizophlyctis rosea	86.1	8.E-16
Rhizophydium globosum	82.4	9.E-15
Rhizophydium globosum	82.4	9.E-15
Rhizophydium laterale	82.4	8.E-15
Rhizophydium sp JEL 385	78.7	9.E-14
Rhizoplaca chrysoleuca	110	2.E-23
Rhizopogon luteolus	654	0.E+00
Rhizopogon luteolus	567	9.E-161
Rhizopogon luteolus	654	0.E+00
Rhizopycnis vagum	87.2	2.E-16
Rhizoscyphus ericae	367	8.E-101
Rhizoscyphus ericae	302	3.E-81
Rhizoscyphus ericae	307	1.E-82
Rhizoscyphus ericae	374	1.E-102
Rhizoscyphus ericae	458	6.E-128
Rhizoscyphus ericae	367	8.E-101
Rhizoscyphus ericae	407	1.E-112
Rhizoscyphus ericae	446	1.E-124
Rhizoscyphus ericae	415	5.E-115
Rhizoscyphus ericae	459	2.E-128
Rhizoscyphus ericae	439	3.E-122
Rhizoscyphus ericae	408	4.E-113
Rhizoscyphus ericae	507	1.E-142
Rhizoscyphus ericae	370	1.E-101
Rhizoscyphus ericae	427	7.E-119

Rhizoscyphus ericae	337	1.E-91
Rhizoscyphus ericae	399	3.E-110
Rhizoscyphus ericae	236	4.E-61
Rhizoscyphus ericae	413	1.E-114
Rhizoscyphus ericae	412	4.E-114
Rhizoscyphus ericae	339	4.E-92
Rhizoscyphus ericae	420	2.E-116
Rhizoscyphus ericae	369	3.E-101
Rhizoscyphus ericae	418	5.E-116
Rhizoscyphus ericae	313	2.E-84
Rhizoscyphus ericae	385	5.E-106
Rhizoscyphus ericae	424	1.E-117
Rhizoscyphus ericae	368	5.E-101
Rhizoscyphus ericae	374	1.E-102
Rhizoscyphus ericae	407	1.E-112
Rhizoscyphus ericae	433	2.E-120
Rhizoscyphus ericae	411	8.E-114
Rhizoscyphus ericae	366	1.E-100
Rhizoscyphus ericae	337	1.E-91
Rhizoscyphus ericae	433	2.E-120
Rhizoscyphus ericae	374	1.E-102
Rhizoscyphus ericae	292	3.E-78
Rhizoscyphus ericae	383	2.E-105
Rhizoscyphus ericae	350	2.E-95
Rhizoscyphus ericae	422	4.E-117
Rhizoscyphus ericae	350	2.E-95
Rhizoscyphus ericae	379	2.E-104
Rhizoscyphus ericae	368	4.E-101
Rhodosporidium babjevae	340	1.E-92
Rhodotorula bloemfonteinensis	329	3.E-89
Rhodotorula bloemfonteinensis	309	4.E-83
Rhodotorula cassiicola	257	1.E-67
Rhodotorula eucalyptica	386	2.E-106
Rhodotorula glutinis	483	2.E-135
Rhodotorula mucilaginosa	578	4.E-164
Rhynchostoma proteae	86.1	7.E-16
Rhynchostoma proteae	86.1	7.E-16
Rhynchostoma proteae	86.1	5.E-16
Rufoplaca tristiuscula	185	6.E-46
Saccharata intermedia	125	1.E-27
Sagenomella diversispora	448	6.E-125
Sagenomella humicola	489	2.E-137
Sagenomella humicola	460	8.E-129
Sagenomella striatispora	462	2.E-129
Sagenomella striatispora	431	9.E-120
Sarcoscypha hosoyae	180	2.E-44
Sarea difformis	163	4.E-39

Sarea resinae	155	6.E-37
Sarea resinae	442	4.E-123
Scedosporium apiospermum	388	5.E-107
Schizothecium glutinans	271	1.E-71
Sclerotium delphinii	63.9	3.E-09
Scolecobasidium terreum	183	2.E-45
Scorias leucadendri	165	6.E-40
Scutellospora calospora	316	2.E-85
Scutellospora calospora	277	1.E-73
Scytalidium vaccinii	316	1.E-85
Sebacina grisea	91.6	1.E-17
Septoria digitalis	305	4.E-82
Septoria escalloniae	222	4.E-57
Septoria lamii	350	2.E-95
Sesquicillium microsporum	418	4.E-116
Sesquicillium microsporum	150	2.E-35
Siphula ceratites	156	3.E-37
Siphula ceratites	156	3.E-37
Siphula ceratites	158	1.E-37
Siphula ceratites	156	4.E-37
Siphula ceratites	183	2.E-45
Sistotrema diademiferum	290	3.E-77
Sistotrema diademiferum	206	5.E-52
Sistotrema sp B216	526	2.E-148
Sistotrema sp B216	490	1.E-137
Sistotrema sp B216	440	1.E-122
Sistotrema sp.	339	3.E-92
Skyttea nitschkei	283	2.E-75
Sordaria fimicola	389	2.E-107
Sordaria fimicola	446	2.E-124
Sordariales sp Pi GPB	261	9.E-69
Sordariomycetes sp 11344	418	5.E-116
Sordariomycetes sp DC2118	467	9.E-131
Sordariomycetes sp DC2118	435	5.E-121
Sordariomycetes sp genotype 106	228	8.E-59
Sorocybe resinae	234	1.E-60
Spadicoides bina	196	3.E-49
Spadicoides bina	212	4.E-54
Sphaerobolus iowensis	569	2.E-161
Sphaeropsis pyriputrescens	326	3.E-88
Sphaeropsis sapinea	464	6.E-130
Spizellomyces acuminatus	152	1.E-35
Spizellomyces dolichospermus	294	8.E-79
Spizellomyces dolichospermus	211	1.E-53
Spizellomyces lactosolyticus	121	2.E-26
Spizellomyces palustris	209	7.E-53
Spizellomyces plurigibbosus	163	3.E-39

Spizellomyces plurigibbosus	102	7.E-21
Spizellomyces pseudodichotomus	264	1.E-69
Spizellomyces pseudodichotomus	183	3.E-45
Spizellomyces pseudodichotomus	93.5	6.E-18
Spizellomyces pseudodichotomus	102	8.E-21
Spizellomyces pseudodichotomus	89.8	7.E-17
Spizellomyces pseudodichotomus	152	7.E-36
Spizellomyces sp JEL 148	80.8	3.E-14
Sporendocladia foliicola	269	3.E-71
Sporobolomyces gracilis	553	2.E-156
Sporobolomyces inositophilus	445	5.E-124
Sporobolomyces lactophilus	290	3.E-77
Sporobolomyces lactophilus	263	3.E-69
Sporobolomyces tsugae	499	3.E-140
Sporormiella sp FBl04	472	3.E-132
Squamarina gypsacea	180	2.E-44
Staphylotrichum boninense	232	2.E-60
Stictis radiata	291	9.E-78
Stictis radiata	282	6.E-75
Stictis radiata	200	2.E-50
Stictis radiata	217	2.E-55
Stictis radiata	241	1.E-62
Stilbum vulgare	123	2.E-27
Strelitziana mali	247	2.E-64
Stropharia cyanea	553	2.E-156
Suillus bovinus	611	5.E-174
Suillus luteus	446	1.E-124
Suillus luteus	611	5.E-174
Suillus luteus	608	4.E-173
Sydowia polyspora	488	6.E-137
Sydowia polyspora	464	6.E-130
Sympodiella acicola	464	8.E-130
Sympodiella acicola	433	2.E-120
Syzygospora bachmannii	291	1.E-77
Syzygospora effibulata	108	1.E-22
Talaromyces verruculosus	374	1.E-102
Talaromyces verruculosus	396	2.E-109
Talaromyces verruculosus	480	1.E-134
Talaromyces wortmannii	480	1.E-134
Teratosphaeria capensis	477	1.E-133
Teratosphaeria capensis	442	4.E-123
Teratosphaeria capensis	453	1.E-126
Teratosphaeria jonkershoekensis	394	8.E-109
Teratosphaeria persoonii	136	3.E-31
Thelephora	581	5.E-165
Thelephora terrestris	431	8.E-120
Thelephora terrestris	577	1.E-163

Thelotrema lepadinum	71.3	1.E-11
Thielavia fragilis	348	5.E-95
Thielavia fragilis	285	4.E-76
Tolypocladium cylindrosporum	451	5.E-126
Tolypocladium inflatum	434	1.E-120
Trechispora hymenocystis	390	1.E-107
Trechispora stevensonii	183	2.E-45
Trechispora stevensonii	418	7.E-116
Trechispora stevensonii	399	2.E-110
Trechispora subsphaerospora	423	2.E-117
Trechispora subsphaerospora	370	1.E-101
Trechispora subsphaerospora	374	1.E-102
Tremella brasiliensis	206	4.E-52
Tremella diploschistina	225	8.E-58
Tremella diploschistina	215	6.E-55
Tremella giraffa	240	2.E-62
Tremella taiwanensis	126	3.E-28
Trichocladium asperum	375	3.E-103
Trichoderma atroviride	38	1.E-01
Trichoderma koningiopsis	481	6.E-135
Trichoderma petersenii	412	4.E-114
Trichomerium deniqulatum	434	1.E-120
Trichomerium gleosporum	331	1.E-89
Trichopezizella otanii	176	3.E-43
Trichosporon debeurmannianum	147	3.E-34
Trichosporon porosum	494	7.E-139
Trichosporon porosum	431	9.E-120
Trichosporon porosum	473	1.E-132
Trichothecium roseum	133	6.E-30
Tricladium chaetocladium	191	9.E-48
Truncatella angustata	462	2.E-129
Tuber cistophilum	85.6	7.E-16
Tuber cistophilum	90.3	3.E-17
Tuber cistophilum	85.6	7.E-16
Umbelopsis autotrophica	548	5.E-155
Umbelopsis autotrophica	536	1.E-151
Umbelopsis isabellina	394	9.E-109
Umbelopsis isabellina	543	1.E-153
Umbelopsis isabellina	357	1.E-97
Umbelopsis isabellina	529	2.E-149
Umbelopsis isabellina	497	7.E-140
Umbelopsis ramanniana	548	5.E-155
Umbelopsis ramanniana	534	5.E-151
Umbelopsis ramanniana	540	1.E-152
Umbelopsis sp I GK 2010	226	2.E-58
Umbilicaria calvescens	218	6.E-56
Umbilicaria calvescens	242	5.E-63

uncultured Archaeospora	488	6.E-137
uncultured Archaeospora	520	1.E-146
uncultured Archaeosporales	470	1.E-131
uncultured Archaeosporales	580	1.E-164
uncultured Archaeosporales	445	4.E-124
uncultured Archaeosporales	507	1.E-142
uncultured Archaeosporales	555	4.E-157
uncultured Archaeosporales	484	5.E-136
uncultured Archaeosporales	NA	NA
uncultured Archaeosporales	531	7.E-150
uncultured Chytridiaceae	52.8	8.E-06
uncultured Cladosporium	446	2.E-124
uncultured Cladosporium	407	1.E-112
uncultured Cladosporium	470	1.E-131
uncultured Dermateaceae	438	4.E-122
uncultured Dermateaceae	399	2.E-110
uncultured ectomycorrhizal fungus	386	2.E-106
uncultured ectomycorrhizal fungus	73.1	6.E-12
uncultured fungus	366	2.E-100
uncultured fungus	353	1.E-96
uncultured fungus	361	6.E-99
uncultured fungus	358	5.E-98
uncultured fungus	196	3.E-49
uncultured fungus	576	1.E-163
uncultured fungus	169	5.E-41
uncultured fungus	391	7.E-108
uncultured fungus	220	2.E-56
uncultured fungus	185	1.E-45
uncultured fungus	569	2.E-161
uncultured fungus	237	1.E-61
uncultured fungus	211	9.E-54
uncultured fungus	418	5.E-116
uncultured fungus	357	1.E-97
uncultured fungus	324	2.E-87
uncultured fungus	292	5.E-78
uncultured fungus	95.3	8.E-19
uncultured fungus	54.7	2.E-06
uncultured fungus	95.3	1.E-18
uncultured fungus	54.7	2.E-06
uncultured fungus	56.5	5.E-07
uncultured fungus	56.5	5.E-07
uncultured fungus	54.7	2.E-06
uncultured fungus	102	5.E-21
uncultured fungus	60.2	6.E-08
uncultured fungus	56.5	5.E-07
uncultured fungus	451	7.E-126
uncultured fungus	556	1.E-157

uncultured fungus	337	2.E-91
uncultured fungus	309	4.E-83
uncultured fungus	95.3	8.E-19
uncultured fungus	339	3.E-92
uncultured fungus	267	1.E-70
uncultured fungus	280	2.E-74
uncultured fungus	421	6.E-117
uncultured fungus	442	4.E-123
uncultured fungus	150	2.E-35
uncultured fungus	664	0.E+00
uncultured fungus	56.5	5.E-07
uncultured fungus	305	6.E-82
uncultured fungus	250	3.E-65
uncultured fungus	298	1.E-79
uncultured fungus	363	2.E-99
uncultured fungus	551	5.E-156
uncultured fungus	87.9	1.E-16
uncultured fungus	158	9.E-38
uncultured fungus	400	8.E-111
uncultured Helotiaceae	418	5.E-116
uncultured Helotiaceae	453	2.E-126
uncultured Hypocreales	87.9	1.E-16
uncultured Leotiomycetes	462	2.E-129
uncultured Leptodontidium	469	3.E-131
uncultured Mortierella	183	3.E-45
uncultured Phaeococcomyces	229	3.E-59
uncultured Phialophora	367	8.E-101
uncultured Sebacina	489	2.E-137
uncultured Sebacina mycobiont of Trifolium pratense	418	5.E-116
uncultured Sebacinaceae	497	7.E-140
uncultured soil fungus	432	3.E-120
uncultured soil fungus	600	1.E-170
uncultured soil fungus	499	3.E-140
uncultured soil fungus	95.1	1.E-18
uncultured soil fungus	481	7.E-135
uncultured soil fungus	115	1.E-24
uncultured soil fungus	93.5	3.E-18
uncultured soil fungus	223	3.E-57
uncultured soil fungus	73.1	6.E-12
uncultured soil fungus	388	6.E-107
uncultured soil fungus	99.9	6.E-20
uncultured soil fungus	610	1.E-173
uncultured Trechisporales	554	6.E-157
uncultured Trechisporales	429	2.E-119
uncultured Trechisporales	547	7.E-155
Unguiculariopsis lettaui	200	2.E-50
Urocystis agropyri	619	2.E-176

Vermispora fusarina	104	2.E-21
Vermispora fusarina	263	4.E-69
Vermispora fusarina	84.2	3.E-15
Verrucaria subcrustosa	231	1.E-59
Verrucariales sp RB 2011	241	1.E-62
Verticillium leptobactrum	518	5.E-146
Wallemia sebi	109	8.E-23
Wallemia sebi	486	2.E-136
Wilcoxina mikolae	420	2.E-116
Wilcoxina mikolae	356	2.E-97
Wilcoxina mikolae	475	4.E-133
Wilcoxina mikolae	470	1.E-131
Wojnowicia sp NW 2013	331	8.E-90
Xanthoria parietina	172	5.E-42
Xenobotrytis acaducospora	250	2.E-65
Xenochalara juniperi	324	6.E-88
Xenochalara juniperi	437	1.E-121
Xenochalara juniperi	379	2.E-104
Xenopolyscytalum pinea	374	1.E-102
Xenopolyscytalum pinea	370	1.E-101
Xenopolyscytalum pinea	416	2.E-115
Xylaria globosa	97.1	3.E-19
Xylaria intracolorata	313	2.E-84
Xylodon sambuci	381	7.E-105
Zeloasperisporium hyphopodioides	213	2.E-54
Zopfiella tabulata	171	2.E-41
Zychaea mexicana	61.8	1.E-08
