# Supplementary material for: Comparison and Validation of Some ITS Primer Pairs Useful for Fungal Metabarcoding Studies
Source: PLoS One. 2014 Jun 16;9(6):e97629. doi: 10.1371/journal.pone.0097629 (PMC4059633; doi:10.1371/journal.pone.0097629)
Supplement: Table S4 — Species used to assess PCR bias of the studied primer pairs (ITS1F/ITS2, ITS3/ITS4 and ITS86F/ITS4). (PDF) [file pone.0097629.s006.pdf]

Supporting Information Table S4

Taxonomic composition of fungal species (n = 15) used in the current study to assess PCR bias at the phylum level

Genus/species	Strain	Family	Order	Phylum
Cladosporium cladosporioides	MUCL 53652	Cladosporiaceae	Capnodiales	Ascomycota
Cryptosporiopsis radicicola	MUCL 53485	Dermateaceae	Helotiales	Ascomycota
Monilinia laxa	MUCL 30841	Sclerotiniaceae	Helotiales	Ascomycota
Arthroderma otae	MUCL 39756	Arthrodermataceae	Onygenales	Ascomycota
Galactomyces geotrichum	MUCL 52377	Dipodascaceae	Saccharomycetales	Ascomycota
Lentinula edodes	MUCL 44827	Omphalotaceae	Agaricales	Basidiomycota
Agrocybe praecox	MUCL 46727	Strophariaceae	Agaricales	Basidiomycota
Coniophora marmorata	MUCL 39471	Coniophoraceae	Boletales	Basidiomycota
Suillus luteus	UH-Slu-Lm8-n1	Suillaceae	Boletales	Basidiomycota
Antrodia vaillantii	MUCL 54533	Fomitopsidaceae	Polyporales	Basidiomycota
Rhizophagus clareus	MUCL 46238	Glomeraceae	Glomerales	Glomeromycota
Rhizophagus sp.	MUCL 41833	Glomeraceae	Glomerales	Glomeromycota
Mortierella verticillata	MUCL 9658	Mortierellaceae	Mortierellales	Zygomycota
Absidia corymbifera	MUCL 38907	Cunninghamellaceae	Mucorales	Zygomycota
Mucor hiemalis	MUCL 15439	Mucoraceae	Mucorales	Zygomycota
