# Supplementary material for: Comparison and Validation of Some ITS Primer Pairs Useful for Fungal Metabarcoding Studies
Source: PLoS One. 2014 Jun 16;9(6):e97629. doi: 10.1371/journal.pone.0097629 (PMC4059633; doi:10.1371/journal.pone.0097629)
Supplement: Table S5 — PCR efficiencies for the amplification of the ITS region of the fungal rRNA operon with the studied primer pairs (ITS1F/ITS2, ITS3/ITS4 and ITS86F/ITS4). (PDF) [file pone.0097629.s007.pdf]

Supporting Information Table S5

PCR efficiencies calculated with qPCR of all species and primer pair combinations used to assess phylum-level PCR bias in the current study

Genus/species	Strain	PCR efficiency (%)		
		ITS1F/ITS2	ITS3/ITS4	ITS86F/ITS4
Cladosporium cladosporioides	MUCL 53652	55	85	92
Cryptosporiopsis radicicola	MUCL 53485	58	56	131
Monilinia laxa	MUCL 30841	74	72	78
Arthroderma otae	MUCL 39756	36	80	91
Galactomyces geotrichum	MUCL 52377	106	87	39
Lentinula edodes	MUCL 44827	70	39	77
Agrocybe praecox	MUCL 46727	73	98	112
Coniophora marmorata	MUCL 39471	73	64	69
Suillus luteus	UH-Slu-Lm8-n1	85	88	94
Antrodia vaillantii	MUCL 54533	51	88	101
Rhizophagus clareus	MUCL 46238	117	126	85
Rhizophagus sp.	MUCL 41833	116	82	74
Mortierella verticillata	MUCL 9658	106	74	86
Absidia corymbifera	MUCL 38907	60	74	65
Mucor hiemalis	MUCL 15439	88	98	93
Average		78	81	86
Standard error		6	5	5
