# Supplementary material for: Comparison and Validation of Some ITS Primer Pairs Useful for Fungal Metabarcoding Studies
Source: PLoS One. 2014 Jun 16;9(6):e97629. doi: 10.1371/journal.pone.0097629 (PMC4059633; doi:10.1371/journal.pone.0097629)
Supplement: Table S6 — PrimerProspector scores of primers used in the current study and investigated by Ihrmark et al. [35] and Toju et al. [36] . (PDF) [file pone.0097629.s008.pdf]

Supporting Information Table S6

PrimerProspector [34] results for all primers investigated in this study and investigated by Ihrmark et al. [35] and Toju et al. [36]

Primer binding site and orientation	Primer name	Primer sequence (5' - 3')	3' mismatch (%)	Average Primer Prospector Score	Standard Error on Score	Reference
5.8S (forward)	ITS86F	GTGAATCATCGAATCTTTGAA	3	0.00	0.00	[27]
	ITS3	GCATCGATGAAGAACGCAGC	4	0.58	0.03	[18]
	gITS9	GAACGCAGCRAAIIGYGA	4	0.41	0.02	[35]
	fITS7	GTGARTCATCGAATCTTTG	4	0.49	0.02	[35]
	gITS7	GTGARTCATCGARTCTTTG	4	0.49	0.02	[35]
	ITS3_KYO1	AHCGATGAAGAACRYAG	5	0.42	0.02	[36]
	ITS3_KYO2	GATGAAGAACGYAGYRAA	2	0.40	0.02	[36]
	58A1F	GCATCGATGAAGAACGC	5	0.51	0.02	[37]
	58A2F	ATCGATGAAGAACGCAG	6	0.52	0.02	[37]
5.8S (reverse)	ITS2	GCTGCGTTCTTCATCGATGC	9	0.70	0.03	[18]
	ITS10mun	GCTGCGTTCTTCATCGAT	3	0.48	0.02	[38]
	ITS2_KYO1	CTRYGTTCTTCATCGDT	2	0.37	0.02	[36]
	ITS2_KYO2	TTYRCTRCGTTCTTCATC	5	0.49	0.02	[36]
	58A2R	CTGCGTTCTTCATCGAT	3	0.47	0.02	[37]

Primer binding site and orientation	Primer name	Primer sequence (5' - 3')	3' mismatch (%)	Average Primer Prospector Score	Standard Error on Score	Reference
28S (reverse)	ITS4B	CAGGAGACTTGTACACGGTCCAG	41	5.75	0.03	[19]
	ITS4_KYO1	TCCTCCGCTTWTTGWTWTGC	23	3.71	0.04	[36]
	ITS4_KYO2	RBTTTCTTTTCCTCCGCT	44	4.39	0.03	[36]
	ITS4_KYO3	CTBTTVCCKCTTCACTCG	42	3.47	0.04	[36]
	ITS4	TCCTCCGCTTATTGATATGC	16	3.96	0.04	[18]
	ITS8mun	CTTCACTCGCCGTTACTA	61	4.07	0.03	[38]
	NL6Amun	CAAGTGCTTCCCTTTCAACA	10	3.49	0.03	[38]
	NL6Bmun	CAAGCGTTTCCCTTTCAACA	9	3.32	0.03	[38]
	NLB3	GGATTCTCACCCTCTATGA	13	3.04	0.03	[37]
	NLB4	GGATTCTCACCCTCTATGAC	53	4.17	0.04	[37]
	NLC2	GAGCTGCATTCCCAAACAACTC	25	3.90	0.04	[37]

Primer binding site and orientation	Primer name	Primer sequence (5' - 3')	3' mismatch (%)	Average Primer Prospector Score	Standard Error on Score	Reference
18S (forward)	ITS1	TCCGTAGGTGAACCTGCGG	12	2.29	0.04	[18]
	ITS1F	CTTGGTCATTTAGAGGAAGTAA	44	4.55	0.05	[19]
	ITS1F_KYO1	CTHGGTCATTTAGAGGAASTAA	36	4.11	0.05	[36]
	ITS1F_KYO2	TAGAGGAAGTAAAAGTCGTAA	15	3.43	0.04	[36]
	ITS5	GGAAGTAAAAGTCGTAACAAGG	8	2.96	0.04	[18]
	ITS9mun	TGTACACACCGCCCGTCG	27	3.34	0.04	[38]
	NSI1	GATTGAATGGCTTAGTGAGG	36	4.11	0.04	[37]
	NSA3	AAACTCTGTCGTGCTGGGGATA	40	4.66	0.04	[37]
